# A genetically defined pontine nucleus essential for ingestion in mice

**DOI:** 10.1073/pnas.2411174122

**Published:** 2025-07-15

**Authors:** Selvee Sungeelee, Caroline Mailhes-Hamon, Zoubida Chettouh, Phillip Bokiniec, Annaliese Eymael, Simon McMullan, Clément Léna, Bowen Dempsey, Jean-François Brunet

**Affiliations:** ^a^Institut de Biologie de l’ENS, Inserm, Centre national de la recherche scientifique, École normale supérieure, Paris Sciences & Lettres Research University, Paris 75005, France; ^b^Queensland Brain Institute, University of Queensland, Brisbane, QLD 4072, Australia; ^c^Faculty of Medicine, Health and Human Sciences, School of medicine, Macquarie University, Macquarie Park, NSW 2109, Australia

**Keywords:** feeding, brainstem, orofacial movements, systems neuroscience, motor systems

## Abstract

The first phase of feeding consists in the procurement of solid foods from the environment by biting, and their preparation for swallowing by chewing. These actions require the precise coordination of tens of orofacial muscles for the jaw and tongue. The seat for this motor patterning is known to reside in the reticular formation, a complex and poorly mapped region of the hindbrain, but the neuron groups involved are still elusive. Here, we characterize a group of excitatory reticular interneurons located in the supratrigeminal area that express the homeodomain transcription factor *Phox2b*. This nucleus—Sup5*^Phox2b^*—is premotor to both jaw-closing and jaw-opening motoneurons and receives direct input from cranial sensory afferents, motor cortex, and satiation related nuclei. Its activity differentially tracks lapping, biting, and chewing movements, suggesting its involvement in the elaboration of distinct orofacial motor patterns in vivo. Acute global activation or inhibition of Sup5*^Phox2b^* by optogenetics interrupt volitional feeding sequences. Thus, Sup5*^Phox2b^* is an obligatory subcortical node, topologically and genetically defined, in the neural circuits that control the oral phase of feeding in mice.

## Significance

We uncover and characterize a group of reticular interneurons in the supratrigeminal area of the hindbrain (termed Sup5*^Phox2b^*). These neurons express the homeodomain transcription factor Phox2b and serve as a premotor nexus for jaw-closing motoneurons, whose activity differentially tracks lapping, biting, and chewing movements. Optogenetic manipulation of Sup5^Phox2b^ disrupts volitional feeding sequences, demonstrating that this nucleus is an essential subcortical node for the control of the oral phase of feeding. This finding advances our understanding of the neural circuits underlying complex orofacial motor behaviors and provides insights into the hindbrain mechanisms that regulate feeding in mice.

The first phase of food ingestion in mammals consists in lapping, or biting followed by chewing. These behaviors are executed by muscles of the jaw and tongue, controlled by motor nuclei in the pons, medulla, and cervical spinal cord. These motor nuclei fall into two genetic classes: i) branchiomotor nuclei for jaw and suprahyoid muscles—trigeminal [principal (Mo5) and accessory (Acc5)], and facial [principal (Mo7) and accessory (Acc7)]; and ii) somatomotor nuclei for the tongue and infrahyoid muscles—hypoglossal (Mo12) and an unnamed cervical motoneuron group (1) that we call MoC (2). The activity of Mo5/Acc5, Mo7/Acc7, Mo12, and MoC is triggered and coordinated at the level of the pons and/or medulla, as evidenced by the preservation of lapping, biting, and chewing in decerebrate or deafferented animals (3–7).

Like all motor circuits, those for feeding can be conceptually parsed into three elements (apart from the motoneurons themselves): a central rhythm generator (for rhythmic movements, such as lapping and chewing); a central pattern generator (that coordinates the muscles involved); and the sensory inputs (that adapt the movements to a changing environment). In practice, the hierarchical level that these circuit modules occupy (presynaptic to motoneurons, or further upstream), and the degree to which they are distinct (the same neurons potentially subserving several functions) is still often speculative.

The circuits for chewing and for lapping—which mobilize many of the same muscles and might overlap [discussed in (8)]—are largely elusive. Proposed locations for a central rhythm generator are based on in vitro *en bloc* preparations (9) and pharmacological infusions of muscimol (10, 11) or lidocaine (12) and are all in the reticular formation, encompassing its intermediate (IRt) and parvocellular (PCRt) divisions mediolaterally, and extending rostro-caudally from Mo5 to the nucleus of the solitary tract (nTS) [reviewed in (13)]. This large region of the hindbrain is also where two types of neurons have been found with relevance to chewing or lapping: i) premotor neurons, most recently mapped by monosynaptic retrograde tracing (2, 14–16); and ii) sensory neurons for the lining of the mouth cavity, periodontal receptors, and jaw-closing muscle spindles, which comprise first-order sensory neurons of the mesencephalic nucleus of the trigeminal nerve (Me5) (15, 16) and second-order sensory neurons of the trigeminal sensory nuclei [reviewed in (17)]. Functional overlap between different neuron types is exemplified by i) premotor neurons that can be construed as part of the central pattern generator, on account of their branching projections to different muscles (2, 15) or to the same muscle bilaterally (14); and ii) second-order sensory neurons in the spinal trigeminal nuclei that are also premotor for Mo5 [reviewed in (17)] and display rhythmic activity during cortically evoked chewing, proposed to reflect an implication in central rhythm generation (18).

Demonstrating, in vivo, a role for any reticular neuron in orofacial feeding movements has been hampered by the dearth of genetic signatures, beyond those that underlie fast neurotransmitter phenotypes. As a consequence, triggering, suppressing, or modulating these movements has been mostly achieved, so far, by relatively unspecific or indirect manipulations of the PCRt: pharmacological interference with GABA_A_ receptors in PCRt/IRt (10, 11, 19); optogenetic and chemogenetic manipulation of all GABAergic PCRt neurons (20); or optogenetic inhibition of inputs to the PCRt from the central amygdala (20); in some cases, movements were triggered together with a more holistic feeding behavior (20), or even with hunger-related depression of thermogenesis (19). To our knowledge, the most direct and specific neuronal manipulations relevant to orofacial feeding movements are those of i) premotor neurons to Mo5 in the supratrigeminal area (Sup5) (optogenetically accessed through their bilateral projections to Mo5, but irrespective of neurotransmitter type), whose excitation and inhibition both increased masseter tone, albeit without triggering or blocking jaw movements (14); and ii) neurons of IRt that express the panvisceral transcription factor *Phox2b* and whose optogenetic excitation triggers lapping (2).

As it happens, *Phox2b* is also expressed in many neurons of the Sup5 area, proposed to contain premotor neurons to Mo5—a century ago using Golgi stains (21), more recently by monosynaptic retrograde tracing (15, 16) (and see above). *Phox2b* thus serves as the first molecular handle on the Sup5 area, and defines the nucleus that we name hereafter Sup5*^Phox2b^*. We show that Sup5*^Phox2b^* targets Mo5, but also Mo12, Mo7, and broad regions of the IRt/PCRt. In alert animals, both photostimulation and photoinhibition of Sup5*^Phox2b^* block bouts of volitional feeding. Moreover, Sup5*^Phox2b^* activity, monitored by fiber photometry, differentially tracks lapping, biting, and chewing, suggesting a role in fine-tuning the orofacial motor output according to feeding modalities.

## Results

### Genetic Signature, Topology, and Development of Sup5*^Phox2b^*.

**Fig. 1.**
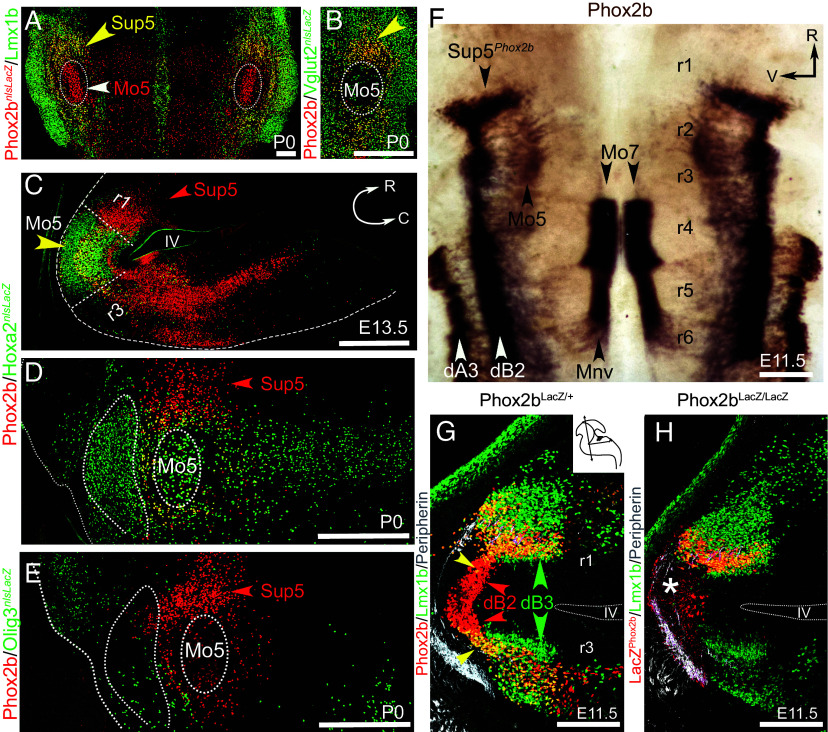
Genetic markers and ontogeny of Sup5*^Phox2b^* (*A* and *B*) Coronal sections at P0 through the pons of a *Phox2b::Cre;Tau-nlsLacZ* (*A*), and a *Vglut2::Cre;Tau-nlsLacZ* mouse (*B*), stained for the indicated markers. (*C* and *D*) Sections through the brainstem of a *Hoxa2::Cre;Tau-nlsLacZ mouse*, parasagittal at embryonic day (*E*) 13.5 (*C*, pontine curvature outlined in white) and coronal at P0 (*D*, principal trigeminal nucleus and Mo5 outlined by stippled line), immunostained for the indicated markers. (*E*) Coronal section through the brainstem of an *Olig3::cre^ERT2^;Tau nlsLacZ* mouse at P0, labeled with the indicated markers. (*F*) Flatmount of a hindbrain at E11.5 embryo hybridized with a *Phox2b* probe [previously analyzed in (25)]. Precursors of Mo7 are still being produced from the pMNv domain of r4 and migrate caudally into r6. Precursors of Mo5 have already migrated dorsally in r2 and settled immediately ventral to the dB2 progeny. Sup5*^Phox2b^* neurons are born in the rostral-most pole of dB2, in r1. (*G* and *H*) Coronal sections (*Inset*) through the pontine flexure of E11.5 embryos, heterozygous (*G*) or homozygous (*H*), for a null *Phox2b* allele, labeled with the indicated markers. The dotted outline indicates the recess of the 4th ventricle, above and below which, r1 and r3 are transversely sectioned, providing mirror images of the progeny of dB2 (Phox2b+) and of dB3 (unlabeled and giving rise to Lmx1b + postmitotic neurons (green arrowheads). The yellow arrowhead indicates the onset of Lmx1b expression in the dB2 progeny (red arrowheads) in r1 (i.e. the prospective Sup5*^Phox2b^*), as well as in r3. In the absence of *Phox2b* (*H*) the dB2 domain does not generate postmitotic neurons (white asterisk in *H*) but postmitotic *Lmx1b* neurons are still observed in r1. IV, fourth ventricle; C, caudal; Mo5, trigeminal motor nucleus; Mo7, facial motor nucleus; MnV, branchiovisceral motoneuronal precursors; R, rostral; r, rhombomere; Sup5, supratrigeminal nucleus; V, ventral. [Scale bars, (*A*), 200 µm; (*B*): 500 µm; (*C*–*E*) 500 µm; (*F*), 400 µm; (*G* and *H*), 200 µm.]

**Fig. 2.**
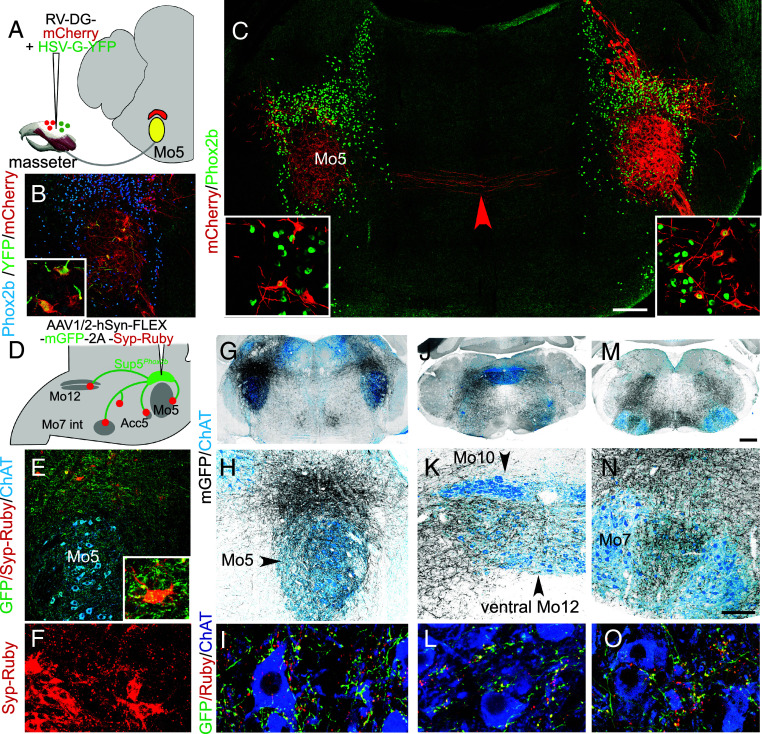
Synaptic targets of Sup5*^Phox2b^*. (*A*–*C*) Monosynaptically restricted labeling of premotor neurons from the masseter in P8 wild-type pups. (*A*) Schematic of the strategy: G-deficient rabies virus (RV) expressing mCherry is coinjected into the masseter along with a YFP-expressing helper virus HSV-YFP-G, providing G-complementation. (*B*) Coronal section through the trigeminal region at P8 (higher magnification as *Inset*), showing mCherry and YFP coinfected “seed cells” in Mo5 (yellow). (*C*) Coronal section through the pons showing premotor neurons (red) that express Phox2b (green) in ipsilateral (right-hand side of the image) and contralateral Sup5 (higher magnification as *Insets*). Red arrowhead: commissural fibers. (*D*–*O*) Anterograde tracing from Sup5*^Phox2b^* neurons. (*D*) Schematic of the injection strategy with summary of the main projection sites. (*E*) Infected neurons in Sup5^*Phox2b*^ express mGFP (green) and syp-Ruby (red). *Inset*: high magnification of an infected neuron in Sup5^*Phox2b*^ itself covered with mGFP+/syp-Ruby+ boutons. (*F*) Dense syp-Ruby puncta from infected Phox2b+ cells in the Sup5 region itself. (*G*–*O*) Coronal sections through the brainstem at low (*G*, *J*, and *M*), and higher (*H*, *K*, *N*) magnifications showing anterograde labeling (gray) of cranial motor nuclei (blue) from Sup5*^Phox2b^*. (*I*, *L*, and *O*) Close-ups of motoneurons in the respective motor nuclei, showing axons (mGFP, green) and terminal boutons (syp-Ruby, red puncta) on the cell bodies. [Scale bar, *G*, *J*, and *M*, 500 µm; *B*, *C*, *E*, *H*, *K*, and *N*, 200 µm.]

**Fig. 3.**
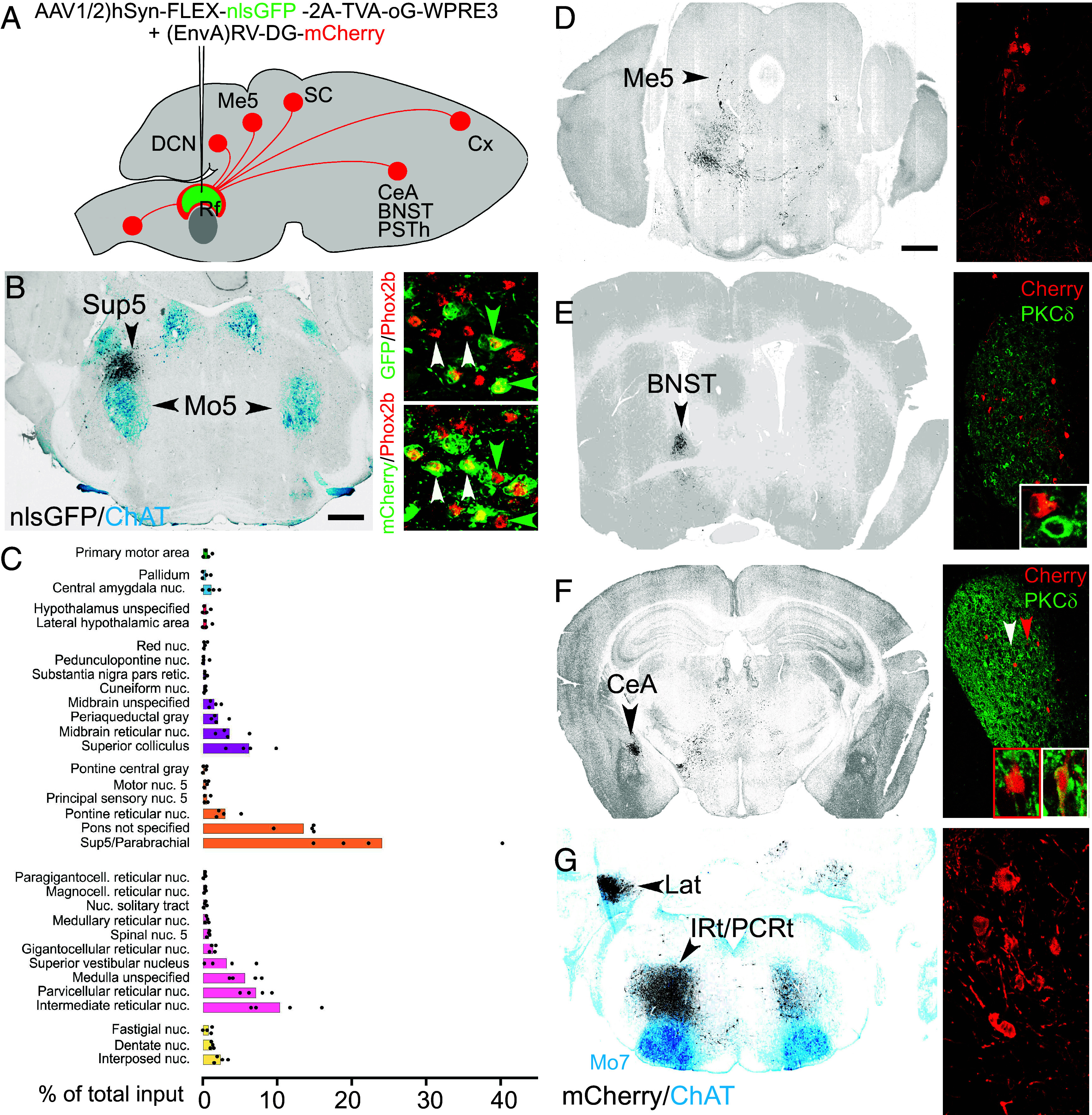
Inputs to Sup5*^Phox2b^*. (*A*) Strategy for monosynaptic retrograde tracing from Sup5*^Phox2b^* and summary of the major inputs. (*B*) Low magnification (*Left* panel) and higher magnifications (*Right* panels) of coronal sections in the Sup5 region. The infected cells of Sup5*^Phox2b^* (black on the *Left* panel) include GFP^+^/mCherry^+^ seed cells (green arrows in *Right* panels) and GPF^−^/mCherry^+^ input cells (white arrows), among which many are Phox2b^+^ (red nuclei). (*C*) Brain-wide proportions of inputs to Sup5*^Phox2b^*. Regions constituting less than 0.3% of total input have been omitted. Note that the contributions of Sup5*^Phox2b^* and parabrachial nuclei are pooled, on account that the “parabrachial” of the Allen Brain Atlas includes a large portion of Sup5*^Phox2b^* as defined in this study. A small fraction of these cells likely do belong to the parabrachial (medial and lateral) as shown in *SI Appendix*, Fig. S3. (*D*–*G*) Other sites of inputs shown on coronal sections showing direct inputs to *^Sup5^*Phox2b*^^* (mCherry+ cells, black in wide views, red in close ups). (*D*) Ipsilateral first-order sensory neurons of the mesencephalic trigeminal nucleus (Me5). (*E* and *F*) The ipsilateral extended amygdala [i.e., the bed nucleus of the stria terminalis (BNST) (*E*) and the central amygdaloid nucleus (CeA) (*F*)]. Most of the inputs from CeA are PKC-∂^−^ (red arrowhead and *Right Inset*) with occasional PKC-∂^+^ inputs (white arrowhead and *Left Inset*); (*G*) the lateral dentate cerebellar nucleus (Lat); the same plane of section captures input from the reticular formation (IRt and PCRt), bilaterally; Other sites of input are in *SI Appendix*, Fig. S3. [Scale bar (applies to all panels): 500 µm (*B* and *G*), 1 mm (*D*–*F*).]

**Fig. 4.**
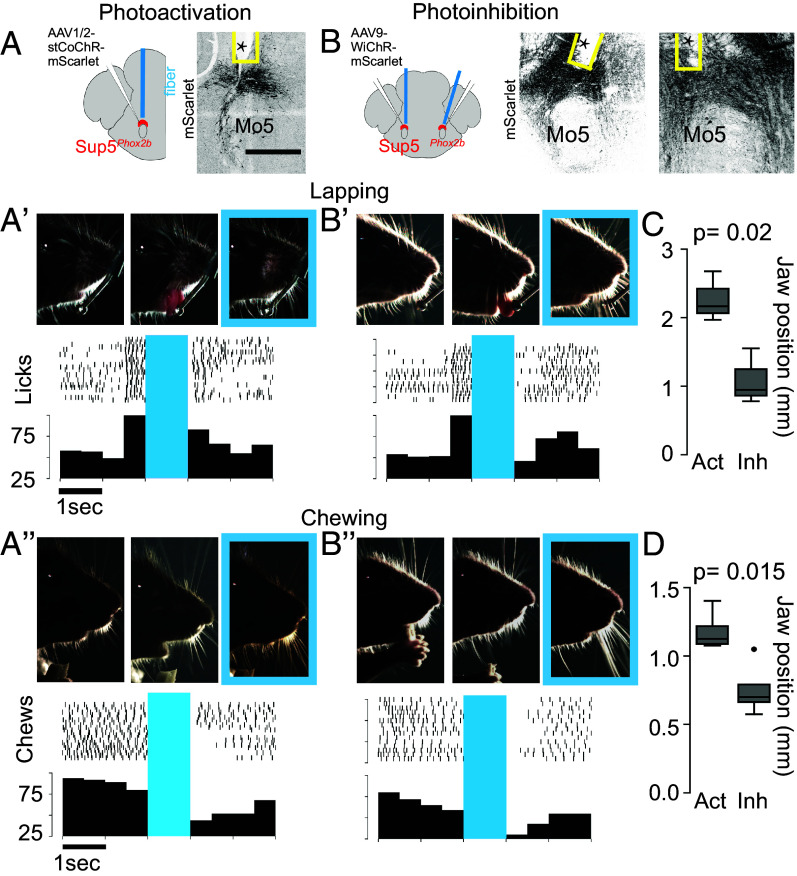
Global activation and inhibition of Sup5*^Phox2b^*. (*A* and *B*) Schematic representation of viral injection in Sup5*^Phox2b^* and fiber-optic implantation, and coronal section of the hindbrain showing infected Sup5*^Phox2b^* neurons and the optic fiber track (asterisk). (*A*′–*B″*) Photoactivation (*A*′ and *A*″) and photoinhibition (*B*′ and *B*″) of Sup5*^Phox2b^* during lapping (*A*′ and *B*′) and chewing (*A*″ and *B*″). Black tick marks indicate jaw movement onsets during chewing or lapping, while histograms show the frequency of licks or chews (n = 15 to 25 trials, n = 3 mice). Blue bars indicate the laser stimulation period and the corresponding photograph is framed in blue. (*C* and *D*) Box-and-whisker plots of jaw displacement caused by activation and inhibition of Sup5*^Phox2b^* during lapping (*C*) and chewing (*D*), relative to the maximal open state during each behavior.

In the adult, Mo5 is surrounded by the reticular formation which can be parsed locally into a “peritrigeminal” area (Peri5) (17) [first described in 1949 as “regio h” on plate XII of (22)]; and a “supratrigeminal” area (Sup5) [first described and named in 1922 by Lorente de No, in Figures 1–4 of (21)]. Both Peri5 and Sup5 are rich in neurons that express the panvisceral homeodomain transcription factor *Phox2b* (2, 23) (and Fig. 1*A*). *Phox2b* can thus be said to define two nuclei in this region: Peri5*^Atoh1^* (that coexpresses *Phox2b* with *Atoh1* and that we previously characterized (2)); and Sup5*^Phox2b^*, the subject of the present study. Like their name implies, Peri5*^Atoh1^* surrounds Mo5, while Sup5*^Phox2b^* caps Mo5, medial to the dorsal aspect of the principal trigeminal nucleus (Fig. 1*A*). Together with *Phox2b*, Sup5*^Phox2b^* expresses the homeobox transcription factor *Lmx1b* (Fig. 1*A*), the vesicular glutamate transporter *VGlut2* (like many other neurons in the surrounding reticular formation, Fig. 1*B*), but not the GABA transporter *GAD67* [present in neurons intermingled with Sup5*^Phox2b^*, *SI Appendix*, Fig. S1*A* and (24)].

Sup5*^Phox2b^* resides—and originates—not dorsal to Mo5 (as its “superior” denomination and position on coronal sections could suggest) but rostral, in topological or geno-architectonic terms (26). This is best seen in a genetic background where a *LacZ* reporter [*Hoxa2::Cre;Rosa^Tau-nlsLacZ^* (27)] labels the second segment of the hindbrain (rhombomere 2): Mo5 is *LacZ*-positive (consistent with its origin mostly in rhombomere 2), while Sup5*^Phox2b^* is *LacZ*-negative and immediately rostral, thus in rhombomere 1 (Fig. 1 *C* and *D*). This topology is also evidenced by the position of Sup5*^Phox2b^* rostral to the Lbx1^+^ interneurons of the medulla, thus to the boundary between rhombomeres 2 and 1 (28) (*SI Appendix*, Fig. S1*B*). Unlike Sup5*^Phox2b^*, most cells of Peri5*^Atoh1^* are derived from rhombomere 2 (Fig. 1*D*) [and see Figure 2*G* in (2)], including those that are contiguous with Sup5*^Phox2b^*. Thus, Peri5*^Atoh1^*and Sup5*^Phox2b^* have distinct developmental origins.

We then mapped the origin of Sup5*^Phox2b^* on the dorsoventral axis of the hindbrain. The *Lmx1b^+^/Phox2b^+^* signature of Sup5*^Phox2b^* (Fig. 1*A*) is shared with derivatives of the pA3 progenitor domain, i.e. dA3 interneurons that comprise the glutamatergic cells of the nucleus of the solitary tract (29–31) and of IRt*^Phox2b^* (2). However, fate tracing in a mouse line that labels derivatives of pA3 (*Olig3::Cre)* reveals that Sup5*^Phox2b^* is not part of them (Fig. 1*E*). Instead, Sup5*^Phox2b^* appears to arise ventrally to pA3, from the pB2 domain, as seen on flatmounts (Fig. 1*F*) and sections (Fig. 1*G*) of the hindbrain at E11.5. And it turns out that pB2 progenitors, known to express *Phox2b* (30), switch on *Lmx1b* postmitotically just like dA3 interneurons do, albeit only in rhombomeres 1 to 3 (arrowheads in Fig. 1*G*), not further caudally [*SI Appendix*, Fig. S1*B* and (31)]. A complication, though, is that the anlage of Sup5*^Phox2b^* extends ventrally, so that it is in register not only with the *Phox2b^+^* pB2 domain, but also with the contiguous *Phox2b^−^* pB3 domain, and is embedded in a broader conglomeration of *Lmx1b^+^* cells that do not express *Phox2b* (Fig. 1*G*). This pattern appears, ambiguously, as resulting either from the postmitotic onset of *Lmx1b* in the *Phox2b*+ progeny of pB2, or the postmitotic onset of *Phox2b* in a subset of the *Lmx1b+* progeny of pB3 (30, 31). An origin in pB2 is supported by the onset of *Lmx1b* in cells migrating out of pB2 (Fig. 1*G*, arrowheads); while an origin in pB3 is supported by the presence of Sup5*^Phox2b^* even in the absence of progeny from pB2, as in *Phox2b* KO (Fig. 1*H*). All in all, despite its anatomical cohesion and its unifying *Phox2b/Lmx1b* signature, Sup5*^Phox2b^* might have a dual pB2/pB3 origin (thus a possible heterogeneity of cell types and functions) that we could not resolve with available tools.

Another ontological landmark of Sup5*^Phox2b^* is that, from its inception, it aggregates around the mesencephalic tract of the trigeminal nerve, i.e. the projections from the mesencephalic nucleus of the trigeminal nerve (Me5) (*SI Appendix*, Fig. S1*C*), which will later provide one of its major inputs (see below).

### Connectivity of Sup5*^Phox2b^*.

The original definition of the Sup5 area—poorly differentiated cytoarchitecturally from the principal trigeminal nucleus laterally, from the reticular formation medially or the parabrachial nucleus rostrally [discussed in (32)]—was mostly based on connectivity: a major target of Me5, and source of input to Mo5 (21). We asked whether these hodological features hold true for Sup5*^Phox2b^*.

First, we assessed whether Sup5*^Phox2b^* is premotor to Mo5 using monosynaptic retrograde tracing from the masseter. We injected the masseter of pups at postnatal day 3 (P3) with a modified G-deficient rabies virus expressing mCherry, together with a G-complementing helper virus expressing YFP, and analyzed brains at P8 (Fig. 2*A*). “Seed cells” coexpressing mCherry and YFP were located in ipsilateral Mo5, more precisely its dorsolateral aspect, consistent with the somatotopic representation of jaw closers in this nucleus (33) (Fig. 2*B*). The Sup5 area (operationally defined as bounded by Me5 rostrally, Mo5 caudally, and coextensive with Phox2b+ cells mediolaterally), contained monosynaptically traced cells, 89% of which expressed *Phox2b* (SD 5.1%, n = 4 animals). Therefore, at least a subset of cells in Sup5*^Phox2b^* are premotor to jaw-closing motoneurons in Mo5. This is in line with previous observations, in transgenic *Phox2b*-GFP rats, of GFP+ cells in Sup5 that project to Mo5 (34). These cells resided in the lateral aspect of Sup5*^Phox2b^*, on both the injected and contralateral sides (Fig. 2 *C*, *Inset*). This lateral bias [not observed in the general population of Sup5 premotor neurons for Mo5 (15, 16)] could reflect the somatotopy of our injection in the masseter (that comprises several fascicles), or the early developmental phase at which the experiment was done (before weaning, and thus presumably before the full engagement of the masseter in feeding).

Next, we evaluated the brain-wide projections of Sup5*^Phox2b^* by injecting it with a Cre-dependent AAV construct that coexpresses a membrane-tethered GFP and a reporter-tagged synaptophysin (Syp-Ruby) that labels synaptic boutons (35) (Fig. 2*D*) (and see all injections sites of this study at the end of *SI Appendix*). The most proximal site of innervation by Sup5*^Phox2b^* was coextensive with Sup5*^Phox2b^* itself (Fig. 2 *E* and *F*). At least some of these synapses were made onto Sup5*^Phox2b^* cells, as evidenced by boutons on the primary infected cells (*Inset* in Fig. 2*E*). In line with the retrograde tracing from the masseter (see above), another dense site of projections was Mo5, which contains jaw adductors (Fig. 2 *G*–*I*); and in line with the necessity to retract the tongue while closing the jaw, dorsal Mo12 [where tongue retractors are located (36)] was also targeted (Fig. 2 *J*–*L*). Thus, like IRt*^Phox2b^* (2) and other groups of orofacial premotor neurons (15, 37), Sup5*^Phox2b^* is in a position to coordinate several orofacial muscles expected to synchronize. Less expectedly, in both cases, boutons were also found in close apposition to the antagonist motoneurons (i.e. in Acc5 that harbors jaw openers (*SI Appendix*, Fig. S2 *A* and *B*), and in ventral Mo12, where tongue protruders have been mapped (36) (Fig. 2*K*), albeit at a much lower density (about 4 and 7 times respectively) (*SI Appendix*, Fig. S2 *B* and *C*), as well as in intermediate Mo7 and accessory 7th nucleus (Acc7) (Fig. 2 *M*–*O*) that contains motoneurons to the platysma (supposedly jaw depressing) and MoC (for jaw opening infrahyoid muscles) (*SI Appendix*, Fig. S2 *D* and *D*′). Projections and boutons also covered the salivary preganglionic neurons dorsal to Mo7 (*SI Appendix*, Fig. S2 *E*-*E*″), a possible substrate for the masticatory-salivary reflex (38). All motor nuclei were targeted bilaterally with ipsilateral predominance.

Other projection sites were, rostral to Sup5*^Phox2b^*, the CeA and BNST (*SI Appendix*, Fig. S2 *F* and *G*), the ventral posteromedial thalamic nucleus (*SI Appendix*, Fig. S2*H*), the deep mesencephalic nucleus (*SI Appendix*, Fig. S2*I*), and caudal to Sup5*^Phox2b^*, the pontine reticular nucleus, caudal part (*SI Appendix*, Fig. S2 *J* and *J*′) and broad regions of the caudal medullary reticular formation (Fig. 2*J*). The remarkably wide range of projections of Sup5*^Phox2b^* suggests either that it controls motoneurons not only directly but also indirectly, or has a more integrative role in feeding behaviors than purely premotor.

Next, we identified the inputs to Sup5*^Phox2b^* by injecting the Sup5 area of *Phox2b::Cre* mice, first with a *cre*-dependent AAV construct expressing the optimized rabies glycoprotein oG, TVA receptor, and nuclear GFP; second, with an EnvA-pseudotyped G-deficient rabies virus encoding the fluorophore mCherry (Fig. 3*A*). Neurons in Sup5*^Phox2b^* that coexpressed both vectors were thus doubly labeled with GFP and mCherry, while their presynaptic partners were single-positive for mCherry (Fig. 3 *A* and *B*, *Right* panels). Consistent with the anterograde projections of Sup5*^Phox2b^* in the Sup5 area (see above), the most proximal (Fig. 3*B*) [and largest (Fig. 3*C*)] collection of presynaptic partners of Sup5*^Phox2b^* was also in this area, comprising other Sup5*^Phox2b^* cells (Fig. 3 *B*, *Right* panels) and possibly other types of cells, yet spatially coextensive with Sup5*^Phox2b^*. Thus, the combined anterograde and retrograde tracings show that Sup5*^Phox2b^* is bidirectionally connected within its own confines, at least in part with other *Phox2b* cells. The second most proximal source of inputs was, as expected from (21), the ipsilateral trigeminal mesencephalic nucleus (Fig. 3*D*). Among other sites of input were, from rostral to caudal: the primary motor and insular cortices (Cx) (*SI Appendix*, Fig. S3 *A* and *B*), the extended amygdala (BNST and CeA) (Fig. 3 *E* and *F*), the parasubthalamic nucleus (PSTh) (*SI Appendix*, Fig. S3*C*), the deep (lateral) superior colliculus (*SI Appendix*, Fig. S3*D*), the lateral (Fig. 3*G*) and other deep cerebellar nuclei (*SI Appendix*, Fig. S3*E*), the parabrachial nuclei (*SI Appendix*, Fig. S3 *F* and *F*′), as well as the IRt and PCRt (Fig. 3*G* and *SI Appendix*, Fig. S3*E*).

### Global Stimulation or Inhibition of Sup5^*Phox2b*^ Prevents Volitional Lapping and Chewing.

To investigate the role of Sup5*^Phox2b^* in vivo, we used optogenetic stimulation. We injected a *Cre*-dependent opsin (39) into the Sup5 area of *Phox2b::Cre* mice, implanted an optic fiber unilaterally above this nucleus (Fig. 4*A*) and video-recorded the faces of head-fixed mice at rest (*SI Appendix*, Fig. S4*A* and Movie S1). Single pulses of light (1,000 ms) triggered an abrupt jaw adduction (of small amplitude since it occurred from the resting, essentially closed, position) in all mice (n = 3) (*SI Appendix*, Fig. S4*A*), with a delay of around 17 ms (suggestive of a disynaptic pathway, and compatible with a premotor status for Sup5*^Phox2b^*), followed by return to the baseline position. Repetitive 100 ms pulses (5 Hz) evoked repetitive jaw adduction from the resting position at the same frequency (Movie S2), showing that Sup5*^Phox2b^* can operate in this frequency range. Thus, Sup5*^Phox2b^* can close the jaw, which is consistent with its projections to jaw closer motoneurons in Mo5. We then asked whether tonic photostimulation of Sup5*^Phox2b^* could disrupt volitional ingestive sequences. We established a closed-loop optogenetic stimulation protocol during lapping in head-fixed mice (*SI Appendix*, Fig. S4*B*), whereby contact between the tongue and a lick-port triggers a 1,000 ms optogenetic stimulation of Sup5*^Phox2b^* after a delay of 480 ms. This stimulation adducted the jaw and abolished all lapping activity (Fig. 4*A*′ and Movie S3 and *SI Appendix*, Fig. S4*B*). Small vertical and horizontal jaw movements occurred immediately after optogenetic stimulation, although lapping per se took longer to resume. This sequence was consistent across all tested animals (n = 3 mice). Similarly, photostimulation of Sup5*^Phox2b^* consistently interrupted the chewing of a flake of almond (n = 4 mice) (Fig. 4*A*″ and *SI Appendix*, Fig. S4*C* and Movie S4).

To investigate whether Sup5*^Phox2b^* is required for orofacial ingestive movements, we resorted to acute optogenetic silencing. We injected WichR1 [a K^+^-selective channelrhodopsin (40)] bilaterally into the Sup5 region of *Phox2b::Cre* mice (Fig. 4*B*) and photoinhibited Sup5*^Phox2b^* tonically for 1 s during periods of rest, lapping, or chewing. At rest, photoinhibition elicited a slight abduction of the jaw from its resting position (suggesting relaxation of jaw adductors), that persisted throughout the stimulation period, and sometimes beyond (*SI Appendix*, Fig. S4*D* and Movie S5). During either lapping (Fig. 4*B*′ and *SI Appendix*, Fig. S4*E* and Movie S6) or chewing (Fig. 4*B*″ and *SI Appendix*, Fig. S4*F* and Movie S7), photoinhibition terminated the ingestive sequence, immobilizing the jaw for the length of the laser signal in a position significantly abducted compared to that caused by photostimulation (Fig. 4 *C* and *D*). Random jaw movements resumed after the end of the laser signal, but it took longer and variable intervals for the animal, possibly disturbed by the interruption, to start eating again. The laser did not produce any movements or interruption thereof in animals injected with a control vector that does not encode an opsin (*SI Appendix*, Fig. S4 *G* and *H*).

Thus, volitional ingestion of liquids or solids cannot proceed during global activation or inhibition of Sup5*^Phox2b^* neurons.

### The Activity of Sup5*^Phox2b^* Tracks Ingestive Orofacial Movements.

**Fig. 5.**
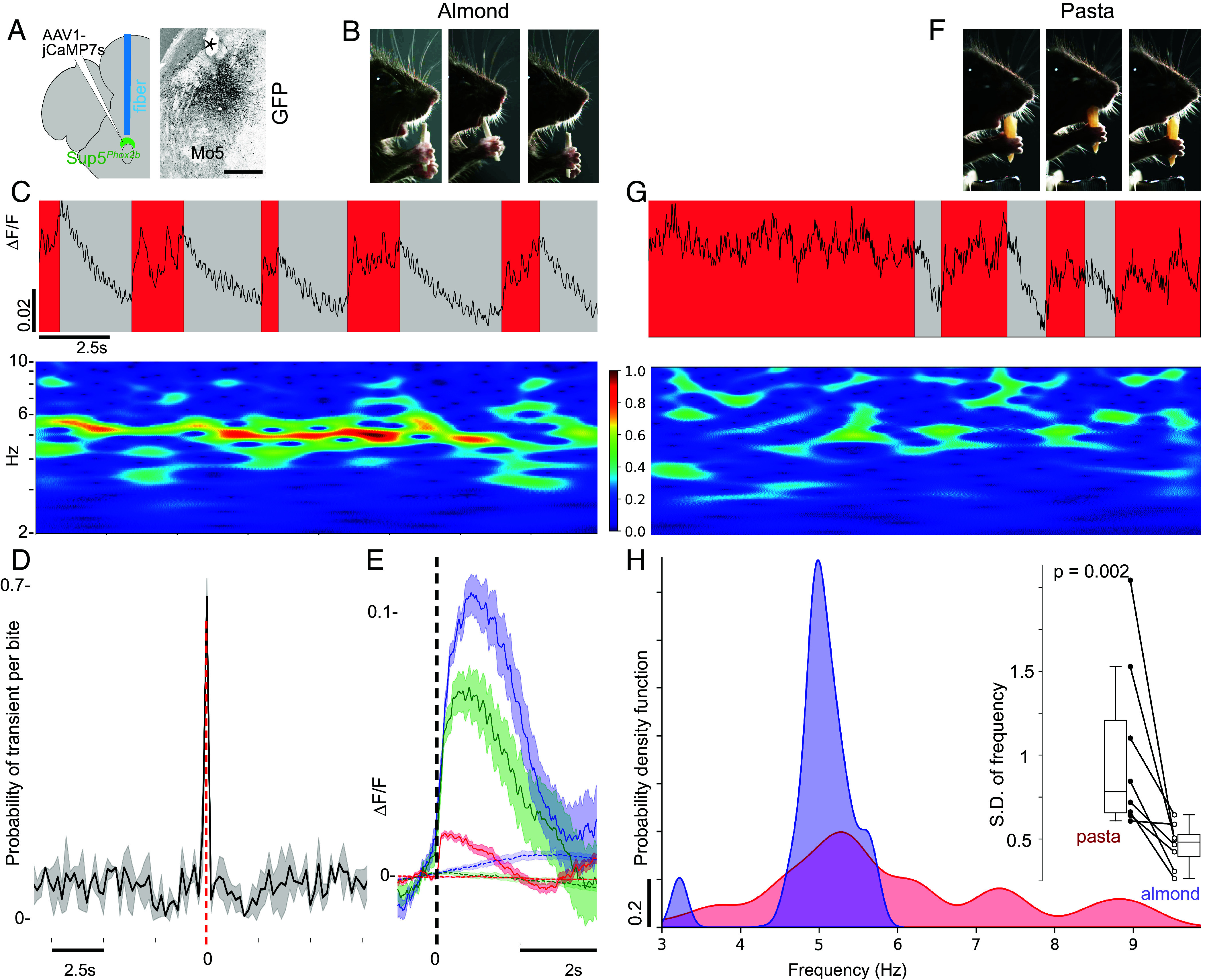
Activity of Sup5*^Phox2b^* during biting and chewing. (*A*) Schematic of viral injection and fiber-optic implantation above Sup5*^Phox2b^*, and transverse section through the hindbrain showing transduced Sup5*^Phox2b^* neurons and the optic fiber track (asterisk). (*B*) Example frames of a mouse during different phases of biting and chewing almond: preparing (*Left*), biting (*Middle*), chewing (*Right*). (*C*) (*Top*) Representative photometry traces of Sup5*^Phox2b^* activity (ΔF/F) over 20 s of chewing almond. Red- and gray-shaded areas indicate biting and chewing epochs, respectively (see *SI Appendix*, Fig. S5 for quantification). (*Bottom*) Continuous wavelet transform of the corresponding photometry signals. Signals around 5 Hz are prominent during almond chewing but diminish during biting. (*D*) Line plot of bite-evoked calcium transients (32 episodes, n = 3 mice): calcium transients were isolated within ±100 ms of 70% of bite onsets (gray shading = SD). (*E*) Event-triggered averages of Sup5*^Phox2b^* activity aligned to bite onset (vertical dashed line) (average of 32 episodes in n = 3 mice, each color represents a different mouse). Shaded area = SEM. (*F*) Example frames of a mouse during different phases of biting and chewing pasta. (*G*) Representative photometry traces of Sup5*^Phox2b^* activity (*Top*) and Continuous wavelet transform (*Bottom*) as in (*C*), but for chewing pasta instead of almond. (*H*) Example kernel density estimate of dominant frequency distribution of the continuous wavelet transform during almond (purple) and pasta (red) consumption. (*Inset*) Dominant frequency variability across conditions (8 sessions, n = 3 mice) (SD of frequency distributions). [Scale bar: (*A*) 500 µm.]

Finally, we tested whether Sup5*^Phox2b^* is active during spontaneous ingestive sequences. We carried out bulk fluorescent calcium recordings of Sup5*^Phox2b^* during spontaneous lapping and biting/chewing in head-fixed mice using fiber photometry (41). We injected a Cre-dependent GCaMP7s vector (42) into the Sup5 area of *Phox2b::Cre* mice and an optical fiber was implanted unilaterally above this nucleus (Fig. 5*A*) to measure the activity of Sup5*^Phox2b^* during jaw movements. In all tested animals (n = 3), lapping bouts correlated with slight increases in Ca2+-induced fluorescence in Sup5*^Phox2b^* (*SI Appendix*, Fig. S5*A*), indicating that Sup5*^Phox2b^* is recruited during lapping. When mice ate an almond flake (Fig. 5*B*), Sup5*^Phox2b^* was also recruited with higher activity compared to lapping (*SI Appendix*, Fig. S5*A*). Moreover, the calcium changes in Sup5*^Phox2b^* tracked the microdynamics of almond ingestion: GCaMP7s fluorescence peaked with each biting event (Fig. 5 *C*–*E*), followed by a slow decay of calcium fluorescence while chewing (Fig. 5 *C* and *E*), during which smaller peaks (ΔF/F = 0.02 ± 0.01) at a frequency of 5.23 ± 0.25 Hz, correlated with each jaw movement (phase difference = −0.008 ± 0.02 s, n = 3 mice) (Fig. 5*C* and *SI Appendix*, Fig. S5 *B* and *C* and Movie S8). These two nested patterns (present in each of n = 8 trials for n = 3 mice) suggest either a switch from tonic to rhythmic activity of Sup5*^Phox2b^*, or the successive operation of two neuronal populations within Sup5*^Phox2b^*, underlying respectively the periodic events of biting and the rhythmic movements of chewing in between. Switching, during the same session, from almond to raw pasta (tougher and less brittle than almond) (Fig. 5*F*), led to a higher frequency of biting events and scarcer and shorter chewing sequences (*SI Appendix*, Fig. S5*D*), that resulted in a more constant activity of Sup5*^Phox2b^*, and the transition from a cyclic alternation of biting and chewing, to a less organized pattern (Fig. 5 *G* and *H*).

All in all, fiber photometry recording of Sup5*^Phox2b^* revealed that its activity differentially tracks orofacial feeding movements. One interpretation is that it parallels the load force (possibly instructed by feedback from Me5): minimal in lapping, highest when biting hard material, and progressively waning during a chewing sequence, as the food is broken down.

## Discussion

We have identified Sup5*^Phox2b^*, a group of glutamatergic neurons marked by the transcription factor *Phox2b*, whose activity tracks jaw movements associated with lapping, biting, and chewing in the alert animal, and whose global optogenetic perturbation, whether by stimulation or inhibition, blocks them. Thus, a minimal functional attribute of Sup5*^Phox2b^* is that of an essential node in the premotor command for food and liquid ingestion. Sup5*^Phox2b^* could, for example, be an obligatory relay downstream of a central rhythm generator. One candidate is IRt*^Phox2b^* [which acts as a lapping central rhythm generator upon photostimulation (2)], with the caveat that we could not ascertain whether the IRt cells that input massively to Sup5*^Phox2b^* (Fig. 3*G* and *SI Appendix*, Fig. S3*E*) express *Phox2b*, thus belong to IRt*^Phox2b^*; if this is the case, IRt*^Phox2b^* could be a common central rhythm generator for chewing and lapping [as was proposed on account that the two behaviors involve many of the same muscles (8)], downstream of which Sup5*^Phox2b^* would implement a chewing/biting pattern, shaped by the jaw proprioceptors in Me5 (17) (Fig. 3*D*); one could even speculate that Sup5*^Phox2b^*, by virtue of its reciprocal connections with IRt, could partake in rhythm generation by providing cyclical feedback to IRt*^Phox2b^*. On the other hand, the fact that Sup5*^Phox2b^* produced no rhythmic jaw movements upon photoactivation seems to exclude prima facie the possibility that it is a rhythm generator by and of itself. However, optogenetic activation of a putative rhythm generator might be a poor method for triggering rhythmic activity, and we are not aware of any published case so far—except, as it were, for IRt*^Phox2b^* and lapping (2): It could be that, in many excitation paradigms, the tonic bulk photoactivation of all potentially rhythmic cells prevents their rhythmic oscillations. Arguing that Sup5*^Phox2b^* should not, in future studies, be written off as an orofacial central rhythm generator, or part thereof, are that i) it resides in the minimal block of tissue that can produce, in vitro, a 4 to 8 Hz rhythm in the trigeminal nerve, or attached masseter and digastric muscles: “between the caudal pole of Mo5 and within 1 mm rostral to Mo5” (9, 43); ii) it receives and sends many connections within its own confines (Figs. 2 *E* and *F* and 3*B*), suggesting the existence of a microcircuitry, amplifying or self-limiting, classically invoked to underlie pacemaker networks in vertebrates (44, 45).

Other afferents to Sup5*^Phox2b^* place it in the position to integrate influences from higher centers, a striking proportion of which are associated with regulation of food intake: the central amygdala (46), the parasubthalamic nucleus (47–49), the bed nucleus of the stria terminalis [(50) and references within], and the lateral deep cerebellar nucleus (51). In turn, several of these sites are targets of Sup5*^Phox2b^* (CeA and BNST) (Fig. 3 *E* and *F*). It is not straightforward to predict the combined effect of these inputs, based on their documented properties: The CeA cells that are presynaptic to Sup5*^Phox2b^* are presumably GABAergic (52), which could fit with an inhibitory role in ingestive behaviors, but for the most part do not express PKC-∂ (Fig. 3 *E* and *F*, *Right* panels), i.e. are not the PKC-∂^+^ cells most clearly implicated, so far, in anorexia (46, 53). As for the PSTh, it is considered glutamatergic (54), and thus should activate Sup5*^Phox2b^*, not expected from an anorexigenic center (although global stimulation could lead to functional suppression, similar to the effect obtained with optogenetics). Whatever the integrated effects of these inputs, one can predict from their documented anorexigenic roles, combined with the requirement for Sup5*^Phox2b^* in ingestion, that it is negative; and one can hypothesize that it is preferentially on biting, i.e. on the initiation of food intake, rather than chewing, a fairly stereotypical action whose only useful modulation should come from oral sensations. In line with this, the activity of Sup5*^Phox2b^*, as measured by fiber photometry, is maximum during biting (Fig. 5 *C*–*E*). On the other hand, even though mastication movements do not make sense, indeed never occur in the context of feeding before food has entered the mouth, a recruitment of the premotor neurons by higher orexigenic centers (e.g. in a predatory context as in (20)), or their inhibition by anorexigenic centers (as suggested by the connectome of Sup5*^Phox2b^*) can be rationalized as the “priming” or “gating,” respectively, of an activity before it is required (55).

**Fig. 6.**
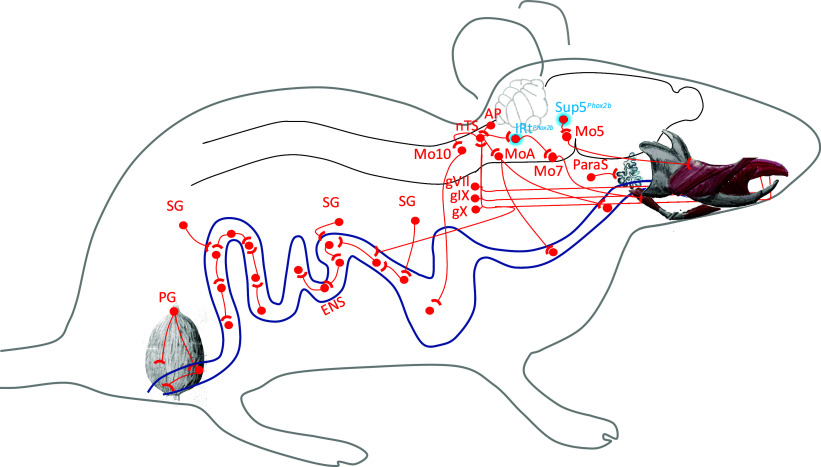
Schematic of the “extended enteric nervous system” of vertebrates. Schematic of a mouse with *Phox2b*-expressing neurons relevant for feeding (represented in red) including two orofacial premotor centers (highlighted in blue). AP: area postrema (involved in vomiting and conditioned food aversion); ENS: enteric nervous system; gVII, gIX, gX: geniculate, glossopharyngeal, and vagal ganglia for all visceral sensations, including taste; IRt*^Phox2b^*: Phox2b+ neuron group in the intermediate reticular nucleus (that harbors a lapping center). MoA: nucleus ambiguus (involved in esophageal mobility); Mo7: facial motor nucleus (that mobilizes orofacial muscles); Mo10: dorsal motor nucleus of the vagus nerve (preganglionic to the enteric nervous system); nTS: nucleus of the solitary tract (site of projection of all visceral afferents, including taste); ParaS: parasympathetic ganglia for salivary glands; PG: pelvic ganglion (that innervates pelvic organs including rectum and bladder, the latter shown in gray); SG: sympathetic ganglia (that input to the enteric nervous system); Sup5^*Phox2b*^, supratrigeminal nucleus (essential promotor center for food ingestion, this study).

The expression of *Phox2b* in a lapping center [IRt*^Phox2b^* (2)], as well as an orofacial premotor center essential for ingestion (Sup5*^Phox2b^*, this study), expands the physiological correlate of this developmental transcription factor (Fig. 6). *Phox2b* was originally discovered as a master gene for the “autonomic nervous system” as defined by Langley (56), i.e. for every neuron type of the autonomic outflow, including the enteric nervous system (with the exception of sympathetic preganglionics) (23, 57); It later emerged that the developmental role of *Phox2b* correlates with a more holistic “visceral nervous system” (58) that includes the sensory afferents to the autonomic outflow (cranial visceral sensory neurons, including those for taste, and their projection site, the nucleus of the solitary tract) (59); as well as the branchiomotor neurons (or “special visceromotor neurons”) (60) to orofacial muscles, whose ancestral roles are exclusively in feeding and breathing. Our previous (2) and current characterizations of IRt*^Phox2b^*, Peri5*^Atoh1^*, and Sup5*^Phox2b^* reveal that, upstream of *Phox2b*^+^ branchiomotor neurons, yet another layer of *Phox2b* neurons is involved in triggering and patterning their activity during lapping, biting, and chewing (and presumably suckling). Thus, the landscape of *Phox2b* neurons, while transcending classical neuroanatomical entities (and crossing the border between peripheral and central, as well as between “autonomic” and voluntary), outlines a novel ensemble, strikingly coherent from a physiological standpoint, which could be coined an “extended enteric nervous system:” the circuits that control all nutritive actions, from the capture of nutrients or fluids from the environment to the rejection of waste, through appetitive and aversive taste perception, salivation or other digestive secretions, and movements of the alimentary tract. Somehow, it was evolutionarily expedient to recruit, over and over again, the same neuronal transcription determinant, *Phox2b*, for elaborating the outer and inner behaviors that provide the cells of the body with fluids and calories. The functional survey of *Phox2b* neurons is nearing completion but some remain to be investigated, e.g. derivatives of the pB2 domain caudal to Mo5 (Fig. 1 *C* and *F* and *SI Appendix*, Fig. S1 *B* and *C*). It is an intriguing possibility that they will also partake in such behaviors.

## Materials and Methods

### Mouse Lines.

The following transgenic mouse lines were used in this study: *Phox2b::Cre* (61), *vGlut2::Cre* (62), *Tau::Syp-GFP-nlsLacz* (also known as *Tau-mGFP*) (63)*, HoxA2::Cre* (27) *Phox2b:LacZ* (64), *Olig3::CreERT2* (31). All mouse lines were bred on a B6D2 F2 background. The experiments were performed on embryos at embryonic (E) days E9.5 to 17.5, neonate pups at postnatal day 2 to 8 (P2–8), and adult (P30–56) animals of either sex. All experimental procedures and protocols were approved by the Ethical Committee CEEA-005 Charles Darwin (authorization 26763-2020022718161012) and conducted under EU Directive 2010/63/EU. All possible measures were taken to minimize the suffering and number of animals used. The sequence of all primers for genotyping are in Table 1.

**Table 1.** sequences of oligonucleotides used for genotyping

Phox2b::Cre	Forward 5′-GGC CGG TCA TTT TTA TGA TC-3′ Reverse 5′ -GAA ATC AGT GCG TTC GAA CGC TAG
Vglut2::Cre	Forward 5′-TGA TGG ACA TGT TCA GGG ATC-3′ Reverse 5′-GAA ATC AGT GCG TTC GAA CGC TAG-3′
Olig3::Creert2	Forward 5′-TGA TGG ACA TGT TCA GGG ATC-3′ Reverse 5′-GAA ATC AGT GCG TTC GAA CGC TAG-3′
Tau :: Syp - GFP	LacZ Forward 5′-AGT TCA CCC GTG CAC CGC-3′ LacZ Reverse 5′-CGC TCG GGA AGA CGT ACG-3′ Tau WT Forward 5′-ATG CGG TAC CTC TTT GGT GCT GTCC CTG C-3′ TAU WT Reverse 5′-CAG ACT GTG CTC CAC TGT G-3′
HoxA2::Cre	Forward 5′-TGA TGG ACA TGT TCA GGG ATC-3′ Reverse 5′-GAA ATC AGT GCG TTC GAA CGC TAG-3′
Phox2b::LacZ/LacZ	LacZ Forward 5′-AGT TCA CCC GTG CAC CGC-3′ LacZ Reverse 5′-CGC TCG GGA AGA CGT ACG-3′ Phox2b Forward 5′-AGT_GGC CCT TCA CAT CCT CA-3′ Phox2b Reverse 5′- AGG CTG CGC AAC TGT TGG G-3′

### Animal Husbandry and Housing.

Mice were housed in groups in conventional housing conditions, at 21 to 22 °C, 40 to 50% humidity, with unlimited access to standard chow and water and kept in a 12-h light/dark cycle.

Mice that underwent surgical implantation for optogenetics and photometry experiments were subjected to a 12-h reverse light/dark cycle and tested in the dark phase.

### Tamoxifen Injections and Cre Recombination.

Cre-recombination in *Olig3-CreERT2* crosses at E9.5 was induced by intraperitoneal injections of 50 mg/kg body weight of tamoxifen in corn oil in pregnant dams.

### Viral Vectors for Tracing, Optogenetic, and Photometry Experiments.

Anterograde tracing from Sup5*^Phox2b^* was carried out by unilateral injection, 3- 4-mo old *Phox2b::Cre* mice, of 250 nl of a Cre-dependent vector (pAAV-hSyn-FLEX-mGFP-2A-Synaptophysin-mRuby, titer: 7 × 10^12^ viral genomes (vg)/ml, Addgene #71760-AAV1) which expresses membrane localized GFP in neurons and a red fluorescent reporter mRuby fused to the synaptophysin gene, which accumulates in the synaptic boutons, thereby outlining their synaptic targets (35)

To map the inputs to the masseter muscle, 50 to 100 nl of a 1:1 viral cocktail of RV-B19-ΔG-mCherry (titer: 1.3 × 10^9^ transduction units (TU)/ml, Viral Vector Core—Salk Institute for Biological Studies) and an HSV-hCMV-YFP-TVA-B19G (titer: 3 × 10^8^ TU/ml, Viral Core MIT McGovern Institute) was unilaterally injected into the masseter of mouse pups (P3).

To label inputs to Sup5*^Phox2b^* a two-step tracing strategy was used (65, 66): First, 50 nl of a Cre-dependent AAV1/2-Syn-flex-nGToG-WPRE3 (titer: 8.1 × 10^11^vg/ml, Viral Core Facility Charité) was injected into the Sup5 of *Phox2b::Cre* mice. This vector expresses the genes for an EnvA-interacting receptor (TVA), a nuclear localized green fluorescent protein (nls-GFP), and an optimized rabies SADB19G protein (oG) (65). Two weeks later, 100 nl of EnvA-pseudotyped rabies virus (EnvA-RABV-SADB19-ΔG-mCherry; titer: 3.1 × 10^8^ vg/ml, Viral Vector Core, Salk Institute for Biological Studies) was injected at the same coordinates. The rabies virus specifically infects AAV-containing, thus TVA+ neurons (seed cells), which subsequently enable its transport in presynaptic neurons by their expression of oG. Thus, seed cells express both nls-GFP and m-Cherry, presynaptic cells express m-Cherry alone. After an additional 7 d, the brains were harvested and the presynaptic neurons were identified based on their location in relation to the Paxinos Atlas (67). Mapping was done using the Allen Brain Atlas 2015.

For photoactivation experiments, Sup5*^Phox2b^* of *Phox2b::Cre* mice was unilaterally transduced by AAV1/2-Ef1a-DIO-stCoChR-P2A-mScarlet (250 nl, titer: 3 × 10^13^vg/ml, kind gift from O. Yizhar) that drives expression of a soma-targeted opsin.

Acute silencing of Sup5*^Phox2b^* was achieved by bilateral infection with a AAV-CAG-flex-WiChR-TS-mScarlet-ER-WPRE3 (250 nl, titer: 0.8 × 10^12^vg/ml, Viral Core Facility Charité) that encodes a K + -selective soma-targeted opsin.

AAV-Ef1a-DIO mScarlet was injected (250 nl, titer: 1.2 × 10^12^ vg/ml, Addgene #131002-AAV1) into Sup5*^Phox2b^* as a control for optogenetic experiments.

For photometry experiments, a unilateral 250 nl injection of AAV1-syn-FLEX-jGCaMP7s-WPRE (titer: 1 × 1,012 vg/ml, Addgene #104487-AAV1) was used to express GCaMP7s in Sup5*^Phox2b^*.

### Surgical Procedures.

#### Stereotaxic injections and implants.

*Phox2b::Cre* mice were anesthetized with an intraperitoneal injection of 50 mg/kg Zoletil (Zoletil 100, Virbac Sante Animale, France) and 10 mg/kg Xylazine (Rompun, 2%). Thirty minutes before the start of surgery, buprenorphine (0.3 mg/ml, 0.1 mg per kg body weight, Buprecare) was administered subcutaneously as analgesia. Core temperature was maintained within the physiological range using a homeothermic pad (37.5 to 38 °C). Briefly, anesthetized animals were placed in a stereotaxic frame (Kopf), and 100 μl of lidocaine (2%) was injected under the skin overlying the skull for local analgesia. To target Sup5*^Phox2b^* neurons, standard surgery was performed to expose the brain surface above Sup5 at the following stereotaxic coordinates: bregma −5.00 mm, lateral ±1.40 mm, dura −2.80 mm.

For viral injections, 50 to 250 nl volumes were delivered at the rate of 75-100nl per minute via quartz glass capillary needles (QF100-50-7,5, WPI) backfilled with mineral oil with a 10μL Hamilton syringe (701 RN) connected to a pump (Legato 130, KD Scientific, Phymep, France). After infusion, the injection needle was maintained in position for 5 min to reduce backflow of the virus during needle retraction. For optogenetics experiments, 200 μm core optic fibers (0.39 NA, Smart Laser Co., Ltd) were placed at the same dorsoventral coordinates as the injections. For photometry experiments, optic fibers (0.57 NA, Smart Laser Co., Ltd) were implanted 200 μm below the injection sites (Dura −3.00 mm). An anchor screw was then placed into the skull. anterior to the brainstem. The optic fibers were subsequently secured by UV-cured dental adhesive cement (Tetric Evoflow, Ivoclar Vivadent) applied to the skull, ceramic ferrule, and anchor screw. Custom-made head-fixation implants (Ymetry, Paris France) were positioned onto the skull and similarly affixed with dental adhesive cement. For inhibition experiments, that require bilateral optic-fiber placement, one of the optic fibers was implanted at an angle of 5° above Sup5 (axis of rotation at ±1.40 mm lateral from Bregma) to leave space for the head-bar on the skull between the two implants. The angled coordinates for the placement of the optic fiber to target Sup5*^Phox2b^*, calculated by basic trigonometry, were bregma −5.00 mm, lateral ±1.73 mm, and skull −3.81 mm.

Mice recovered from anesthesia on a heating pad for a day before being placed back into their home cages. Appropriate postsurgical care was provided, and animals were regularly monitored for signs of infection, pain, or lethargy until behavioral assays began.

#### Intramuscular masseter injections.

Masseter muscle injections were performed at P3 neonatal stage. Pups were anesthetized by deep hypothermia. For anesthesia induction, pups were placed in latex sleeves and gently submerged in crushed ice for 3 to 5 min. Anesthesia was maintained (up to 10 min) by placing pups on a cold pack (3 to 4 °C). A small incision was made in the skin overlaying the masseter and a glass pipette (tip diameter ca. 0.1 mm), filled with the virus cocktail and connected to a pneumatic dispenser (Picospritzer), was guided into the masseter with a 3D micromanipulator. Muscular filling with the viral cocktail was achieved with 5 to 10 pressure pulses (100 ms, 3 to 5 bars), which delivered around 50nl of the virus. This was confirmed by the spreading of Fast-Green dye (0.025%) added to the viral solution. After injection, the pipette was withdrawn, and the incision was irrigated with physiological saline before suturing [10-0 gage suture (Ethilon)]. Pups were promptly returned to the mother after a brief recovery onto a heating pad. The brains were harvested five days after injection (P8) for histology.

### Histology.

#### Immunofluorescence.

Depending on the stage, histology of the brain was carried out either on whole embryos (up to E16.5) dissected out of uterine horns or on brains dissected out of the cranial vault for embryos E17.5 to P0. Adults and postnatal animals were killed by an intraperitoneal injection of pentobarbital (Euthasol Vet, 140 mg/kg), transcardially perfused with ice-cold PBS followed by 4% PFA in PBS (Antigenfix, Diapath), and the brains were rapidly dissected.

Brains or embryos were then postfixed in 4% PFA overnight at 4 °C, rinsed 3 times for 30 min each in PBS then cryoprotected in 15% sucrose in PBS, overnight at 4 °C. Tissues were subsequently embedded in gelatin-sucrose medium (7.5% gelatin in 15% sucrose in PBS) and frozen for cryosectioning at 30-60 μm on a CM3050s cryostat (Leica). Sections were washed for 1 h in PBS and incubated in a blocking solution (10% calf serum in 0.5% Triton-X100 PBS) containing the primary antibody, which was applied to the surface of each slide (300 μL per slide). Slides were placed in a humidified chamber on a rotating platform and incubation lasted 4 to 8 h at room temperature and overnight at 4 °C. Sections were washed in PBS (3 × 10 min), then incubated in the dark with the secondary antibody in a blocking solution for 2 h at room temperature. Following PBS washes (3 × 10 min), slides were air-dried and mounted under a coverslip with a fluorescence mounting medium (Dako, #S3023).

Primary antibodies used were goat anti-Phox2b (1:100; RD system, AF4940,), rabbit anti-peripherin (1:1000; Abcam,ab4666), guinea pig anti-Lmx1b [1:1,000; (68)], goat anti-ChAT (1:100; Millipore, AB144p), chicken anti-βGal (1:1,000; Abcam, ab9361), chicken anti-GFP (1:1,000; Aves Labs, GFP-1020), rabbit anti-Lmx1b [1:2,000; (68)], guinea pig anti-Lbx1 [1: 10,000; (68), rabbit anti-DsRed (1:500; Takara bio, 632496), rabbit anti-PKCdelta (1:1,000; Abcam,ab182126,), rat anti-RFP(1:500; Rockland, 200-301-379).

All secondary antibodies were used at 1:500 dilution: donkey anti-chicken 488 (Jackson laboratories, 703-545-155), donkey anti-chicken Cy5 (Jackson laboratories, 703-176-155), donkey anti-goat Cy5 (Jackson laboratories, 705-606-147), donkey anti-rabbit 488 (Jackson laboratories, 711-545-152) donkey anti-rabbit Cy5 (Jackson laboratories, 712-165-153), donkey anti-rat Cy3 (Jackson laboratories, 711-495-152), and donkey anti-Guinea pig Cy3 (Jackson laboratories, 706-165-148). DAPI staining (Roth, #6335.1) was used at 1 µg/mL to visualize cell nuclei in some experiments. Epifluorescence images were acquired with a NanoZoomer S210 digital slide scanner (Hamamatsu Photonics) and visualized with the NDPview2+ software; and confocal images with a Leica SP8 confocal microscope (Leica) with Leica Application suite X. Image formatting, including adjustments of brightness and contrast, and pseudocoloring, was carried out in Adobe Photoshop (v.25.5.1) and FIJI.

### Behavioral Experiments.

#### Timing and training.

All behavioral experiments were conducted 3 to 4 wk after viral injections and fiber optic implantation. One week after surgery, the mice were accustomed to handling, head fixation, and tethering to the patch cable during daily 10-min sessions. This consisted in placing them into a plastic cylinder (4 cm diameter) mounted onto a small aluminum breadboard (450 mm × 450 mm × 12.7 mm, ThorLabs; MB4545/M), and head-fixing them using a custom-made fast fixation system (Ymetry, Paris, France) via a skull-attached head fixation implant. Their forepaws rested on the cylinder edge and the head protruded out. Mice were given either a 15% sucrose solution or a piece of almond or pasta in the home cage at the end of each session to reduce neophobia to these substances. During the lapping sessions, mice were given a 15% sucrose solution via a lick port. Chewing epochs were initiated by bringing a piece of almond or a piece of raw pasta within reach of the mouse’s jaw until the mouse grasped it between the jaws and held it in its forepaws. To highlight the jaw silhouette during video acquisitions, mice were illuminated from below and from the sides with white LED lights.

#### Optogenetics and lick-induced photostimulation.

To activate Sup5*^Phox2b^* neurons that express StCochR, a surgically implanted fiber-optic cannula was connected to a 473-nm DPSS laser (CNI, Changchun, China) via a patch cord (200Â μm, 0.39 NA) using a zirconia mating sleeve Thorlabs). Simultaneous bilateral silencing of Sup5*^Phox2b^* expressing WichR1 was achieved using bifurcated fiber bundles (THORLABS BFYL2LS01) connected to the same laser source via an SMA905 connector. Spike 2 software and a 1,401 data acquisition unit (Cambridge Electronic Design) were used to control laser output. Single continuous light pulses of 50 to 1,000 ms or trains of 100 ms pulses at 5 Hz were used. In all behavioral experiments, the minimal laser output required to elicit a response was used, which was measured to be approximately 2 mW at the fiber tip using a digital power meter (PM100USB, Thorlabs).

Laser output was controlled via 5 V pulses from a 1401 data acquisition unit using Spike 2 software (CED Spike 2 Data Acquisition and Analysis Software). For licking paradigms, the laser was triggered mid-bout by a 5 V pulse from a capacitive sensor wired to the lick-port via the 1401 unit with a fixed delay of 480 ms.

#### Photometry.

Single site fiber photometry recordings were made using a Doric fiber photometry system (Doric Lenses Inc, Canada) with 405 nm (isosbestic) and 490 nm excitation using Doric Neuroscience Studio software. Data were acquired at 12 kHz.

#### Automated markerless pose estimation.

Spontaneous and light-evoked oromotor movements were filmed at portrait and profile angles with a CMOS camera (Jai GO-2400-C-USB) synchronized by a 5 V TTL pulse. The acquired frames had a resolution of 800 × 800 pixels and were streamed to a hard disk using 2ndlook software (IO Industries). The frames were then compressed using an MPEG-4 codec at a rate of 120 fps.

A ResNet-50 deep learning model was trained using DeepLabCut (version 2.3.8) on profile frames of mouse faces, to identify the genu of the jaw. This model was used to generate pose estimation of jaw in the experimental videos.

### Data Analysis.

#### Quantification of anterograde tracings.

Images were pseudocolored post hoc using NDP.view2 (Hamamatsu Photonics), with RFP mapped to 645 nm and Cy5 to 444 nm. For three sections per animal, across three animals, Fiji/ImageJ was used to draw regions of interest around subdivisions of Mo5 (Mo5 proper and Acc5), Mo7 (medial, middle, and lateral), and Mo12 (dorsal and ventral) using ChAT expression as cytoarchitectonic guide. The intensity values of red pixels (thresholded to remove background signal while preserving boutons) were summed for each region of interest and divided by the total number of pixels to obtain the average pixel intensity. The corrected intensity signal per ROI was then averaged across the three sections and three animals.

Pixel intensity values were pooled across animals per nucleus (n = 3 mice). For each pairwise comparison between nuclei, the difference in pixel intensity was calculated, and the normality of these difference distributions was assessed using the Shapiro–Wilk test. Depending on the outcome of the normality test, either a paired-samples *t* test or a Wilcoxon signed-rank test was performed, as appropriate, using the scipy.stats library in Python. A significance threshold of *P* < 0.05 was applied for all statistical tests.

#### Quantification of retrograde tracing.

Rabies-labeled inputs to Sup5^*Phox2b*^ neurons were mapped using the QUINT workflow, as previously described (69, 70). In brief, fluorescent images of serial brain sections were scanned and imported into Qupath 0.5.1 software (71). Rabies-labeled neurons were classified as either input neurons (mCherry+) or seed neurons (mCherry+ and GFP+) using the Qupath “positive cell detection” function, and section images and corresponding cell detection maps exported. Section images were initially registered to the Allen Common Coordinate Framework using DeepSlice 1.2.5 (https://github.com/PolarBean/DeepSlice) (72), then manually adjusted to account for nonlinear tissue warping using Visualign 0.9 (RRID: SCR_017978, https://www.nitrc.org/ projects/visualign). The positions of labeled neurons, contained within detection maps, were combined with corresponding registration data using Nutil RRID:SCR_01718355 (73) to assign CCF coordinates to each neuron and count the number of neurons within each region of the 2015 Allen Mouse Brain Atlas. Detections falling outside of the brain, in fiber tracts, or in the ventricular system were eliminated from analysis.

#### Normalization and processing of jaw pose estimation.

The magnitude of jaw movement in pixels was calibrated against the diameter of the fiber optic ferule (1.25 mm) within the video frame. Tracking data for the jaw was smoothed with a Savitzky–Golay filter.

#### Calculation of jaw distance during optogenetic experiments.

Onsets of jaw closure were identified as the peaks of the first derivate of each jaw movement separated from each other by at least 40 ms in a 5 s window during bouts of either lapping or chewing. For both lapping and chewing, jaw distance was calculated as the average position from 50 ms to 500 ms into the optogenetic stimulation window, relative to its maximal open position between −550 ms and −50 ms prior to stimulation.

Data were analyzed using Python (v 3.11.7) and is reported as mean ± sem unless otherwise stated. Statistical significance between photoactivation or inhibition was assessed using a parametric, two-tailed independent Student’s *t* test, following assessments for normality (Shapiro–Wilk test) and equal variance (Levene test). No statistical methods were used to predetermine sample sizes, but our sample sizes were similar to those used in previous publications.

#### Annotation of photometry data during ingestion.

To detect biting versus chewing events during almond or pasta consumption, behavioral videos were annotated by eye using QuickTime Player version 7.7.9 to extract the start and end frames for each of these two behaviors within an 18-s interval. Biting events were characterized as instances when the mouse bit or gnawed the food material to break it into smaller pieces. Chewing events, on the other hand, were defined as the interval between two consecutive biting events, during which the mouse ground the food material between its teeth before swallowing. The resampled and annotated video data were then aligned to the preprocessed photometry data in MATLAB to examine the calcium dynamics during biting and chewing of almond and pasta.

#### Isosbestic signal correction.

The isosbestic control baseline for the photometry signal was estimated as follows: The raw calcium-dependent signal (490 nm) and isosbestic control channels (405 nm) were first preprocessed by applying boxcar averaging and subsampled by a factor of 100 to the frequency 120.48 Hz. The isosbestic line was fitted to the estimated local baseline signal defined by the lower values of the signal during periods of low activity. Briefly, local minima were obtained by first applying a rolling minimum with a window of 5 s (600 samples) followed by selecting the smallest minima when successive minima were closer than 100 samples (830 ms). Only local minima during periods of low activity were kept. The isosbestic line was then adjusted to the baseline signal by estimating an offset and a scale using a linear regression (using Numpy’s polyfit function) of the isosbestic control signal and calcium-dependent signal taken at the time of the local minima. The fluorescence signal was then normalized to calculate ∆F/F, which was generated by subtracting the scaled isosbestic regression (F) from the signal channel and dividing it by F.

#### Probability of calcium transients per bite onset.

The ∆F/F signal was filtered to remove frequencies between 2 and 8 Hz. The following analysis was then performed on the processed signal to detect the onset of transient events in the recorded fluorescence signal. Briefly, the rate of change (“derivative”) at the time scale of ~100 ms was estimated by computing the difference between signal values at delays of 75 to 125 ms (values were adjusted for each session to account for small differences in the rise time). The mean and SD of the first-order difference were computed over the entire signal (80 to 125 s) and transient detection was based on a threshold defined as the mean plus two SD. Time points where the first-order difference crossed this threshold were identified as upward transitions. Due to the time resolution of the GCamp7s signal, events separated by less than 0.4 s were excluded to eliminate spurious detections.

To assess the temporal relationship between transients in photometry recordings and biting onsets that were visually annotated from the behavioral videos, we calculated the temporal offset between each bite onset and transients during a period of 80 to 125 s. To compute the probability of transients per bite onset, the time differences were binned into 200 ms intervals, and their frequency was normalized by the total number of bite onsets. Data were analyzed within a temporal window ranging from -10 to 10 s and a mean histogram across animals was computed, along with the SD.

#### Bite-evoked GCamp dynamics.

We computed event-triggered averages of the ∆F/F signal centered on the onset of biting events (for a duration of 80 to 125 s). The signal was segmented into time windows spanning 1 s before and 4 s after each biting timestamp. Each segment was baseline-corrected by subtracting the mean signal value from the mean prestimulus baseline period (−1 to 0 s relative to stimulus onset) over that trial. The corrected signal segments were then averaged across trials (average of 32 trials per mouse, n = 3 mice), and the SEM was computed. The event-triggered average procedure was then repeated after random resampling of event timestamps within the observed range (n = 100 iterations) to assess variability across sampling iterations and generate a bootstrapped mean response and SEM.

To compare GCaMPs activity dynamics in Sup5*^Phox2b^* neurons during pasta and almond consumption, we analyzed the spectral content of the photometry signal using Continuous Wavelet Transform (CWT), implemented with the PyWavelets Python package. The ΔF/F signal was first high-pass filtered at 3 Hz, and its time-frequency representation was computed using a Morlet wavelet with center frequency and band-width 3.0 (cmor3.0-3.0) within the 3 to 10 Hz range. 20-s segments (n = 8 per condition, from n = 3 mice across 6 sessions) corresponding to consumption periods were transformed using CWT. To ensure comparability, the resulting spectrogram magnitudes were scaled within the global minimum and maximum across both food conditions for each animal. The dominant frequency at each time point was identified and frequencies associated with low wavelet amplitudes (below 40% of the global maximum amplitude for each segment) were discarded. The frequency distribution over each segment of activity studied was then estimated using a Gaussian kernel density estimator with a bandwidth of 0.2. The SD of the dominant frequency distribution was computed for each condition, and statistical comparisons were performed using a linear mixed model (LMM) in SciPy with Condition (Pasta vs. Almond) as a fixed effect, and individual trials as a random effect. The mean peak amplitude and peak frequency of the calcium signal during chewing of almond were averaged for each segment for 8 trials in n = 3 mice and box and whisker plots were generated.

#### Comparison of time spent chewing versus biting.

We compared the fraction of time spent chewing versus biting across food types (pasta or almond), based on timestamps extracted from visual annotations of behavioral videos. All trials (three per animal; n = 9 per condition) were pooled, and matched values across conditions were compared using the Wilcoxon signed-rank test, implemented via the wilcoxon function from the scipy.stats module. All tests were two-sided. Reported *P*-values correspond to each comparison.

## Supplementary Material

Appendix 01 (PDF)

Movie S1. 1000 ms optogenetic activation of Sup5Phox2b at rest.

Movie S2. 5 times 100 ms optogenetic activation of Sup5Phox2b at rest.

Movie S3. 1000 ms optogenetic activation of Sup5Phox2b while licking.

Movie S4. 1000 ms optogenetic activation of Sup5Phox2b while chewing.

Movie S5. 1000 ms optogenetic inhibition of Sup5Phox2b at rest.

Movie S6. 1000 ms optogenetic inhibition of Sup5Phox2b while licking.

Movie S7. 1000 ms optogenetic inhibition of Sup5Phox2b while chewing.

Movie S8. Synchronized DF/F trace and film of mouse biting and chewing an almond.

## Data Availability

All study data are included in the article and/or supporting information.
