# Supplementary material for: A genetically defined pontine nucleus essential for ingestion in mice
Source: Proc Natl Acad Sci U S A. 2025 Jul 15;122(29):e2411174122. doi: 10.1073/pnas.2411174122 (PMC12305073; doi:10.1073/pnas.2411174122)
Supplement: Supplementary file 1 — Appendix 01 (PDF) [file pnas.2411174122.sapp.pdf]

PNAS

Supporting Information for
A GENETICALLY-DEFINED PONTINE NUCLEUS ESSENTIAL FOR INGESTION IN MICE.

Selvee Sungeelee[a], Caroline Mailhes-Hamon[a], Zoubida Chettouh[a], Phillip Bokiniec[b], Annaliese Eymael[c], Simon McMullan[c], Clément Léna[a], Bowen Dempsey[c,1] and Jean-François Brunet[a,1,*]

[a] Institut de Biologie de l'ENS (IBENS), Inserm, CNRS, École normale supérieure, PSL Research University, 75005 Paris, France

[b] Queensland Brain Institute, University of Queensland, St Lucia, Brisbane, QLD, 4072 Australia

[c] Faculty of Medicine, Health & Human Sciences, Macquarie University, Macquarie Park, NSW, 2109 Australia

* Jean-François Brunet

Email : jean-francois.brunet@bio.ens.psl.eu
Phone number: 33 (0) 1 44 32 23 21

This PDF file includes:

Figures S1 to S4
Table 1
Legends for Movies S1 to S4
Supporting Appendix

Other supporting materials for this manuscript include the following:

Movies S1 to S8

[1]B.D and J.F.B contributed equally to this work.

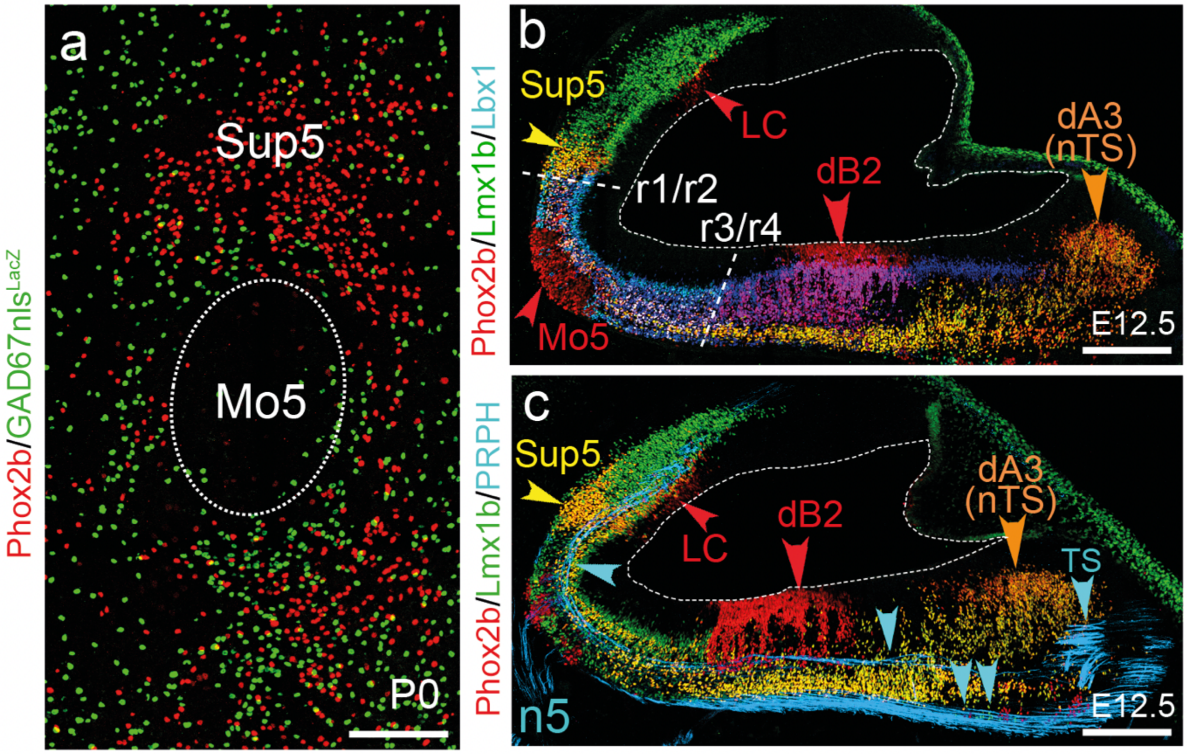

Fig. S1. Genetic markers and ontology of Sup5*Phox2b*.(a) Section through Mo5 and Sup5*Phox2b* at P0 stained for the indicated markers. **(b, c)** Parasagittal sections of the brainstem at E12.5, stained with the indicated markers. The rhomboidal shape of the hindbrain entails that sagittal sections cut through different dorsoventral progenitor domains or their progeny (such as dB2 or dA3) at different rostro-caudal levels. **(b)** Sup5*Phox2b* develops just rostral to the r1/r2 boundary (stippled line), marked by the rostral limit of Lbx1. The r3/r4 boundary (stippled line) is the caudal limit of Lmx1b expression in Phox2b+ /Lbx1+ dB2 progeny (triple-labeled cells, white), caudal to which they are Phox2b+ / Lbx1+ / Lmx1b — (purple). **(c)** The emerging Sup5*Phox2b* lies close to the Me5 tract, which runs all the way to the caudal hindbrain (blue arrow). LC, locus coeruleus; n5, trigeminal nerve; nTS, nucleus of the solitary tract; PRPH, peripherin; TS solitary tract. Double blue arrow: spinal trigeminal tract; Sup5: Sup5*Phox2b*. The fourth ventricle is outlined with a stippled line.

.

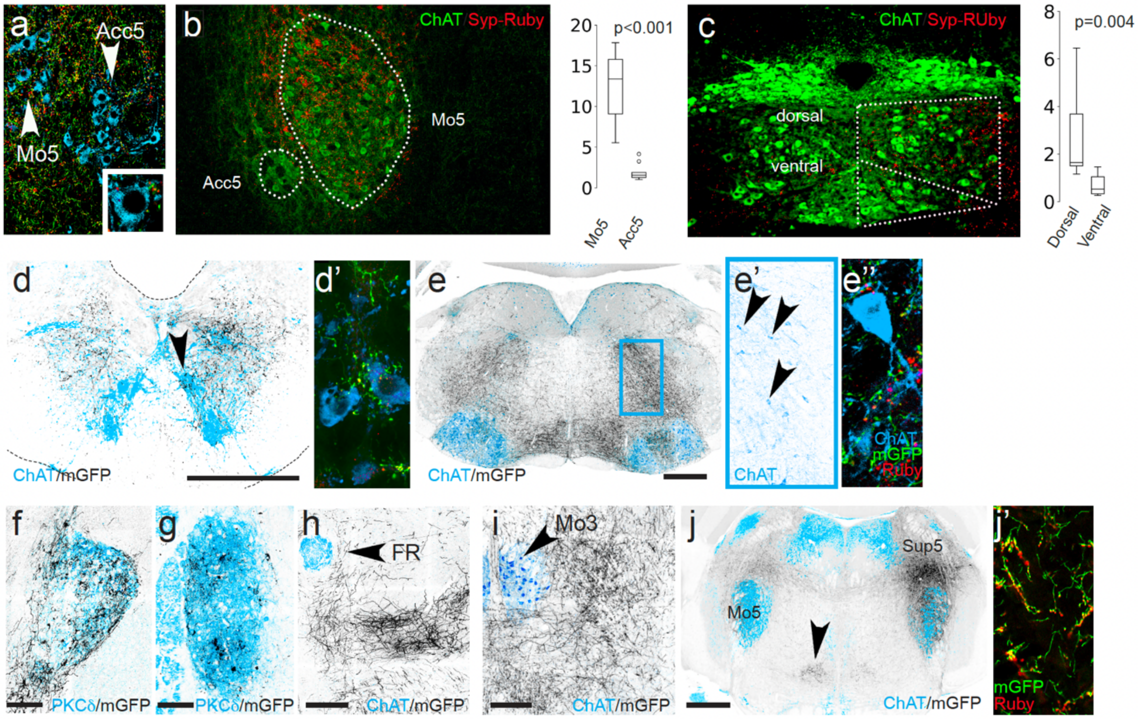

Fig. S2. Additional sites of projections of Sup5^{Phox2b}.(**a**) Section through Mo5 and Acc5 showing mGFP+ fibers and syp-Ruby+ boutons from Sup5^{Phox2b} on both nuclei. Inset: close up of a neuron in Acc5. (**b**,**c**) cytoarchitectonic delineation of Mo5 (**b**) and Mo12 (**c**) used to compare the density of boutons in Mo5 versus Acc5 and in the dorsal versus ventral compartments of rostral Mo12, respectively, and related counts (arbitrary units). **(d,d')** Projections of Sup5^{Phox2b} on MoC (arrowhead in (**d**), high magnification in (**b'**). Blue: ChAT; green: mGFP; red:syp-Ruby.(**e**,**e'**,**e''**) The inferior salivatory nucleus located in the ventrolateral reticular formation receives projections (grey) on both sides (**e**), with ipsilateral predominance. (**e'**) Magnification of the blue boxed region in (**e**), showing the characteristically dispersed ChAT+ cells of the inferior salivatory nucleus in the reticular formation above Mo7 (arrowheads). (**c''**) Close-up showing a ChAT+ salivatory nucleus cell covered with syp-Ruby boutons.(**f**-**i**) More rostral regions targeted by the Sup5^{Phox2b} include the extended amygdala (central amygdala (**f**) and the bed nucleus of the stria terminalis (**g**)); the ventral posteromedial thalamus (**h**) and the deep mesencephalic nucleus surrounding Mo3 (**i**).(**j**,**j'**) The caudal pontine reticular nucleus (arrowhead) located at the rostrocaudal level of Mo5 and Sup5 (**j**) receives dense inputs from the Sup5^{Phox2b}. (**j'**) Higher magnification showing Syp-Ruby puncta in the pontine reticular nucleus. BNST: bed nucleus of the stria terminalis; CeA: central amygdala; DPME: Deep mesencephalic nucleus; FR: Fasciculus Retroflexus; Mo3: oculomotor nucleus, PnC: Pontine reticular nucleus caudalis; VPM: ventral posteromedial thalamus. Scale bars, **d**-**e''** 500 μm; **f**-**g** 200 μm; **h**-**j** 100 μm.

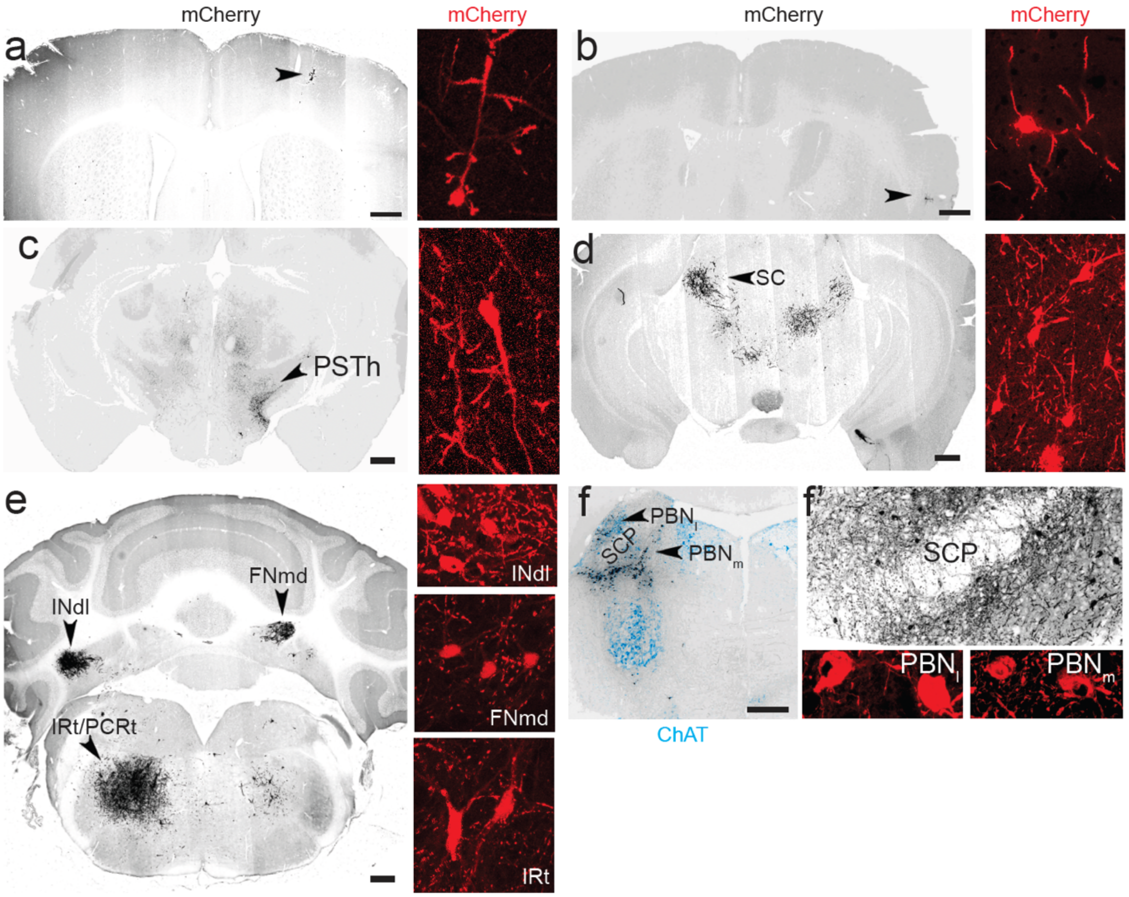

Fig. S3. Additional sites of inputs to Sup5*Phox2b*. Inputs (mCherry+) are shown at low magnification (grey, left panels) and high magnification (red, right panels). **(a,b)**, motor **(a)** and insular **(b)** cortices provide contralateral input; (**c**) Posterior subthalamic nucleus (PSTh) (**d**) superior colliculus (SC) — more specifically its intermediate and deep lateral (motor-related) layers — projects ipsilaterally to Sup5*Phox2b*; (**e**) in addition to Lat (**Fig. 3**) several deep cerebellar nuclei target Sup5*Phox2b*, including the dorsolateral interposed nucleus (INdl) (ipsilaterally) and medial dorsolateral fastigial nucleus (FNmd) (contralaterally); (**f**, **f'**) parabrachial nucleus (PBN) (medial and lateral divisions). SCP: superior cerebellar peduncle. Scale bars: 500 µm.

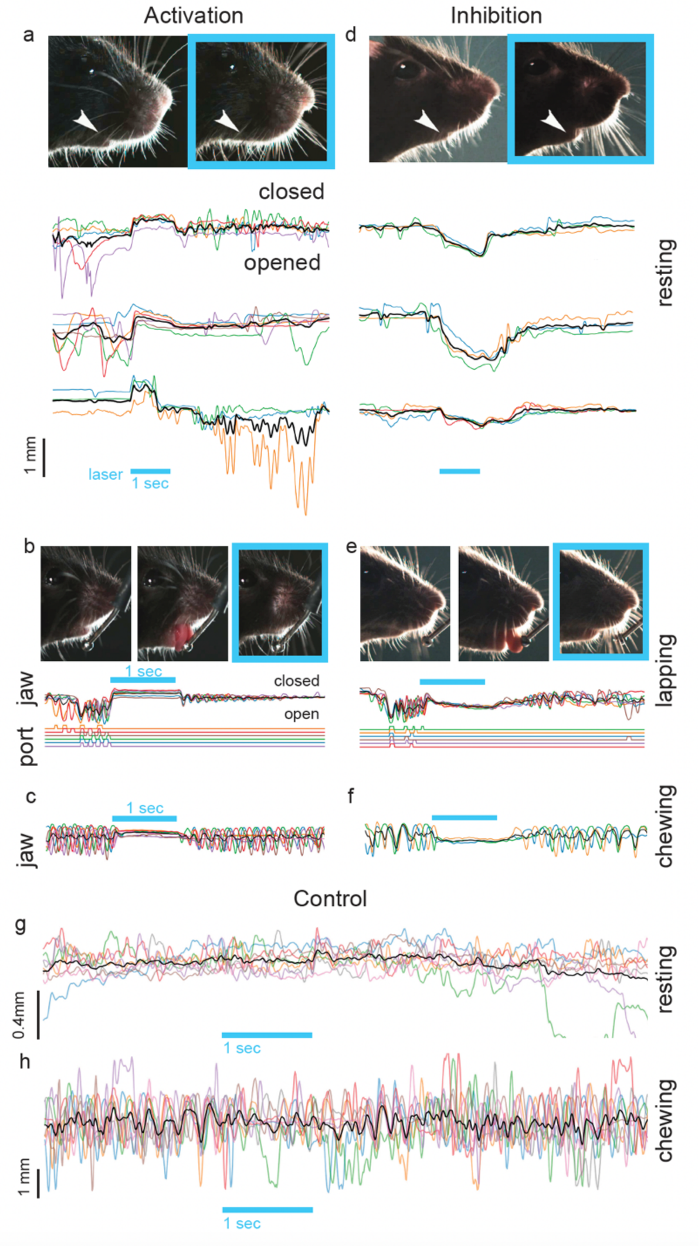

Fig. S4 Individual traces of jaw movements during activation and inhibition of Sup5*^{Phox2b}*. (**a**) Close-up of the face of a mouse before and during (blue frame) a 1 s activation period, and individual traces (3 to 5 trials each in n=3 mice, grand average in black). (**d**) Equivalent panels for inhibition. Stimulation induces a slight upward jaw movement, throughout the stimulation period, or only part of it depending on mice; inhibition induces a slight downward movement. (**b**,**e**) Close-up of the face of a mouse lapping (preparing (**left**), licking (**middle**), during laser stimulation (**right**)) and individual traces below (5 trials for one mouse). (**c**,**f**) Equivalent traces for chewing. (**g**,**h**) Control traces of 1 animal injected with a conditional mScarlet-encoding vector in Sup5^{Phox2b}, stimulated by a 1 s laser pulse, during rest (**g**) or chewing (**h**). Jaw movements and position are unaffected by the laser pulse. A second control showed the same lack of effect.

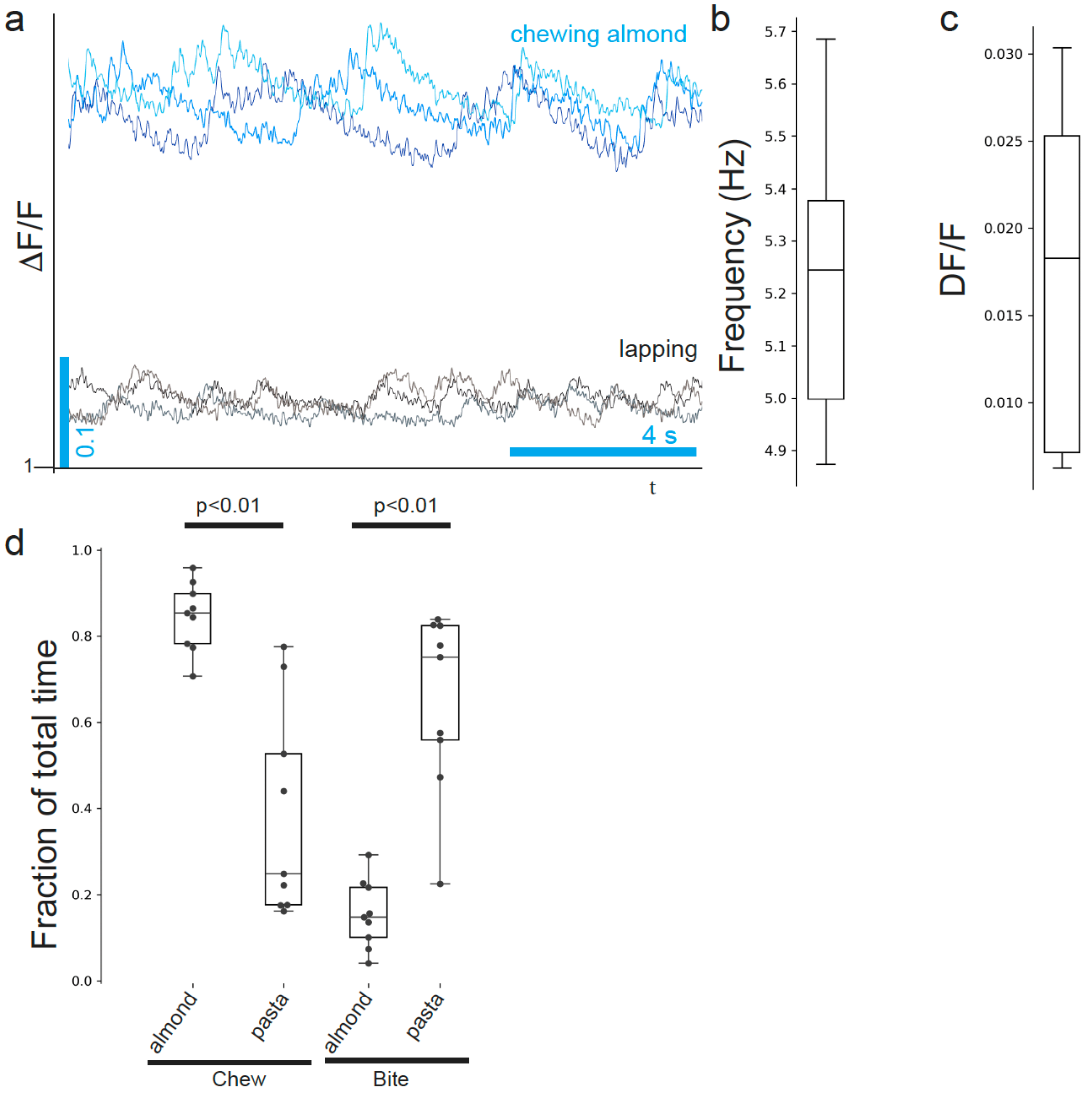

Fig. S5. Related to Figure 5. (**a**) ΔF/F comparison between lapping (3 lower traces) and chewing almond (3 upper traces) in 3 trials on one mouse. (**b**,**c**) Box and whisker plots of dominant frequencies (**b**) and of amplitudes (**c**) extracted from the CWT of the photometry signal during chewing epochs when eating an almond (n=8 trial, n=3 mice). (**d**) Box and whisker plots of time spent chewing versus biting, almond or pasta.

Phox2b::Cre	Forward 5'-GGC CGG TCA TTT TTA TGA TC-3'<br>Reverse 5' -GAA ATC AGT GCG TTC GAA CGC TAG
Vglut2::Cre	Forward 5'-TGA TGG ACA TGT TCA GGG ATC-3'<br>Reverse 5'-GAA ATC AGT GCG TTC GAA CGC TAG-3'
Olig3::Creert2	Forward 5'-TGA TGG ACA TGT TCA GGG ATC-3'<br>Reverse 5'-GAA ATC AGT GCG TTC GAA CGC TAG-3'
Tau :: Syp - GFP	LacZ Forward 5'-AGT TCA CCC GTG CAC CGC-3'<br>LacZ Reverse 5'-CGC TCG GGA AGA CGT ACG-3'<br>Tau WT Forward 5'-ATG CGG TAC CTC TTT GGT GCT GTCC CTG C-3'<br>TAU WT Reverse 5'-CAG ACT GTG CTC CAC TGT G-3'
HoxA2::Cre	Forward 5'-TGA TGG ACA TGT TCA GGG ATC-3'<br>Reverse 5'-GAA ATC AGT GCG TTC GAA CGC TAG-3'
Phox2b::LacZ/LacZ	LacZ Forward 5'-AGT TCA CCC GTG CAC CGC-3'<br>LacZ Reverse 5'-CGC TCG GGA AGA CGT ACG-3'<br>Phox2b Forward 5'-AGT_GGC CCT TCA CAT CCT CA-3'<br>Phox2b Reverse 5'- AGG CTG CGC AAC TGT TGG G-3'

Table1: sequences of oligonucleotides used for genotyping

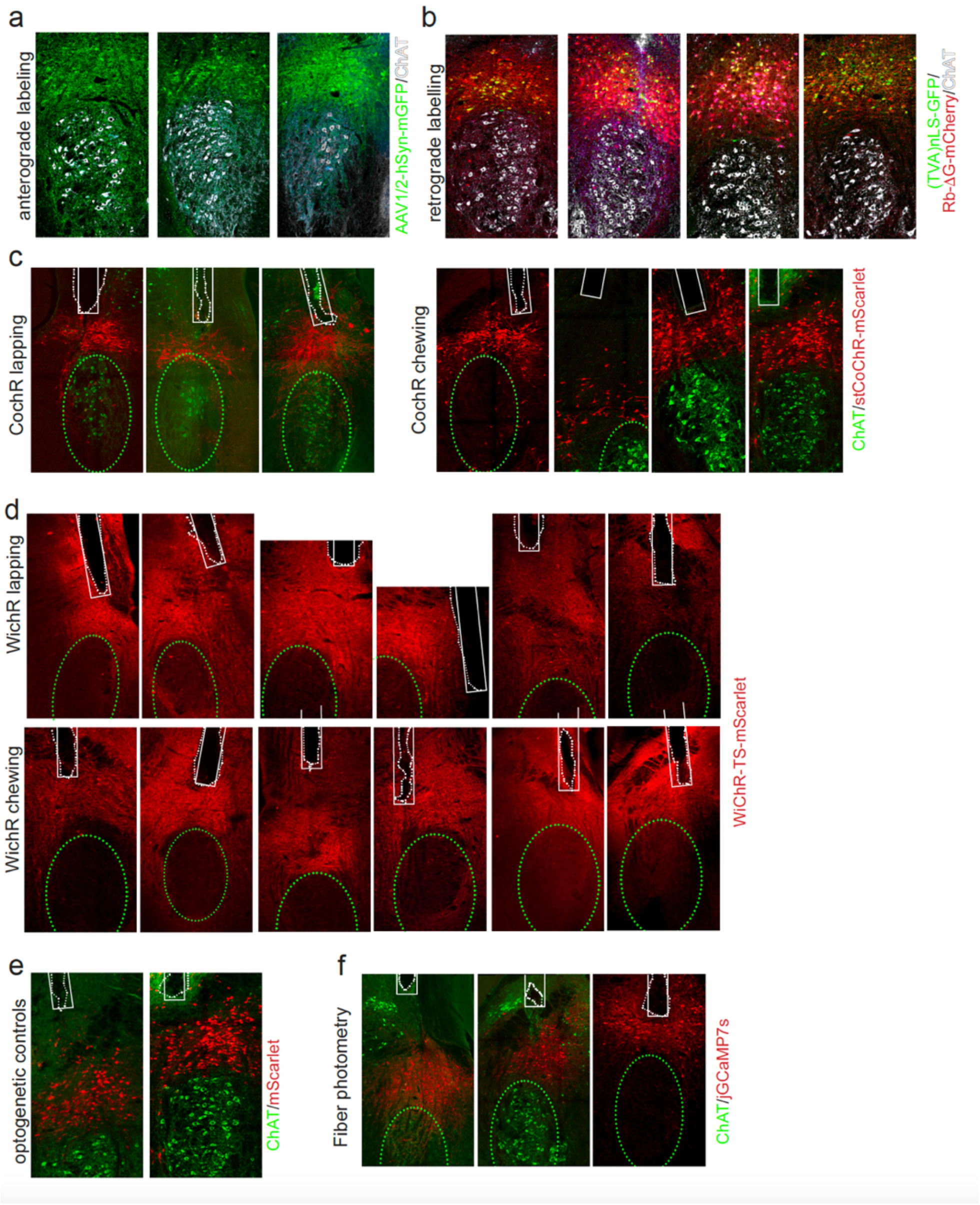

Supporting Appendix. Injections sites for all tracing and physiological experiments. (a) Seed cells (green) for anterograde tracing; (b) Seed cells (yellow) for retrograde tracing; (c) Infected cells (red) and fiber position (plain line) inferred from borders of tissue tearing (stippled line) for photoactivation experiments. (d) Same as (c) for bilateral photoinhibition experiments. In mouse GA839 the left fiber was misplaced, yet inhibition worked as well as for the other mice. (e) Same as (c) for photometric recording. ChAT labeling was not performed for WichR experiments and did not work for mice FT570 and FG203. Note that WichR labeling, although theoretically somatically targeted (1), massively labels fibers in our hands.

Movie S1 (separate file). 1000 ms optogenetic activation of Sup5Phox2b at rest

Movie S2 (separate file). 5 times 100 ms optogenetic activation of Sup5Phox2b at rest

Movie S3 (separate file). 1000 ms optogenetic activation of Sup5Phox2b while licking

Movie S4 (separate file). 1000 ms optogenetic activation of Sup5Phox2b while chewing

Movie S5 (separate file). 1000 ms optogenetic inhibition of Sup5Phox2b at rest

Movie S6 (separate file). 1000 ms optogenetic inhibition of Sup5Phox2b while licking

Movie S7 (separate file). 1000 ms optogenetic inhibition of Sup5Phox2b while chewing

Movie S8 (separate file). Synchronized DF/F trace and film of mouse biting and chewing an almond.

REFERENCES

1. A. Forli, M. Pisoni, Y. Printz, O. Yizhar, T. Fellin, Optogenetic strategies for high-efficiency all-optical interrogation using blue-light-sensitive opsins. *eLife* **10**, e63359 (2021).
